# tvsfglasso: Time-varying scale-free graphical lasso for network estimation from time-series data

**DOI:** 10.1371/journal.pcbi.1013710

**Published:** 2025-11-17

**Authors:** Markku Kuismin, Mikko J. Sillanpää

**Affiliations:** Research Unit of Mathematical Sciences, University of Oulu, Oulu, Finland; Xinjiang Technical Institute of Physics and Chemistry, CHINA

## Abstract

In high-dimensional gene co-expression network analysis, capturing the temporal changes of gene associations is crucial for unveiling dynamic regulatory mechanisms inherent in biological systems. Examining how these interactions change over time offers valuable insights into the developmental and adaptive processes that drive an organism’s lifecycle. Moreover, incorporating structural prior information can substantially enhance the accuracy and interpretability of the estimated sparse dynamic gene network. Methods previously proposed in the literature cannot simultaneously model sparse time-varying co-expression network structure and have the power-law degree distribution. Additionally, there is a demand of time-efficient, memory-light software implementations and possibility to utilize repeated measures at each time-point (if available). In this paper, we introduce the time-varying scale-free graphical lasso (tvsfglasso), a novel scalable framework for estimating high-dimensional time-varying gene co-expression networks under the assumption that these networks simultaneously exhibit sparse and a scale-free structure. We utilize fast algorithms developed for the graphical lasso (glasso), which makes tvsfglasso a scalable tool for high-dimensional problems. We evaluate the performance of tvsfglasso using both simulated and real-world dynamic gene expression time series datasets, demonstrating its capability to detect temporal changes in gene associations. Our results highlight the potential of tvsfglasso to advance the understanding of dynamic gene networks, making this estimator useful for more accurate modeling of complex biological processes.

## Author summary

Dynamic co-expression network analysis can provide insights into processes such as development, disease progression, and treatment response. However, although power-law distributions are frequently used to model co-expression networks characterized by a few hub genes and many genes with few connections, currently there are no tools to construct this type of networks from high-dimensional time-series data. We propose the time-varying scale-free graphical lasso (tvsfglasso) to investigate gene interactions over time in complex biological systems when a scale-free network is used to model gene co-expression topology. The method utilizes fast algorithms from the literature that enable the construction of dynamic gene networks with thousands of genes and only a handful of observations. We show in simulations that tvsfglasso can capture subtle changes in dynamic gene networks in high accuracy. Applied to *Drosophila melanogaster* embryo time-series data, it revealed bursts of new regulatory links just before key developmental transitions. This work delivers a powerful tool for constructing networks from high-dimensional time-series gene expression data.

## 1 Introduction

Gene co-expression network analysis of large-scale multivariate expression data is widely used to understand how genes interact with each other in the same biological process [see, e.g., [1–4], for reviews]. For recent advances in using various network-based tools, such as protein–protein interaction networks and graph neural networks, combined with large language models (LLMs), for cancer gene identification, drug-drug interaction prediction, and protein complex analysis, see [5–7]. Gene co-expressions can be presented as an undirected network, where each node represents a specific gene and edges describe pairwise conditional dependencies between genes. Under a Gaussian assumption, edges correspond to nonzero entries of the precision matrix (the inverse of the covariance matrix). This framework, known as the Gaussian graphical model (GGM), provides an elegant way to remove linear confounding effects while examining pairwise relationships between variables.

Well-studied and time-efficient methods suitable for high-dimensional network analysis such as the graphical lasso (glasso) [8,9] have been employed to construct sparse time-invariant GGMs. However, in many practical settings, one might be interested in examining the relationships between genes across time points [see [10], for a review]. Dynamic behavior is observed in biological systems, where gene co-expression patterns can vary during different developmental stages or in response to external stimuli. In these cases, inferring a single static network may obscure critical transient interactions and lead to misleading conclusions.

Time-varying network analysis seeks to capture these evolving relationships by allowing the underlying graph topology to change over time. This problem, however, introduces remarkable challenges: First, the network may undergo various types of changes, from abrupt shifts involving many nodes to more subtle edge-wise adjustments. Second, capturing the underlying dynamics of regulatory systems is difficult unless expression are measured on a slowly changing system [1,10]. Third, methods designed for static inference struggle to accommodate the increased complexity and computational demands imposed by sparse and high-dimensional time-varying networks [see, e.g., [11]. Previous approaches utilizing the glasso estimator have attempted to address these challenges by imposing smoothness on the sample covariance matrices of neighboring time points [12] or by employing penalties, which encourage piecewise constant changes in the network topology [11] or by applying a local group-lasso type penalty [13] or scaled Lasso [14]. See [15] for a glasso variant tailored to high-dimensional data with multiple observation classes. While these methods have advanced our ability to analyze time-varying associations, they are computationally demanding. Furthermore, software tools designed for time-varying network estimation are scarce.

Here we introduce a new time-varying glasso estimator. This is a novel approach designed for analyzing gene co-expression networks from time series data. Because gene co-expression networks are often observed to have scale-free properties [16,17], we incorporate *a priori* information about the scale-free structure by imposing a power-law constraint on the degree distribution, thereby guiding the network topology. This *a priori* information about the network structure can lead to more informative conclusions. This new method not only facilitates the interpretation of the network’s evolution but also improves the estimator’s sensitivity in detecting biologically meaningful variations.

Rather than introducing completely new algorithms and procedures, we combine ideas from the methods proposed earlier in the literature. In particular, we (i) use the adaptive scale-free penalty proposed by [18] to add extra prior information to our gene network inference task; (ii) combine it with the time-varying covariance matrix smoothing procedure proposed by [12] [see also [13]; and (iii) propose a modification of the smoothing procedure of [12]. Our approach generalizes the estimator proposed by [12] for setting where several biological replicates have been collected for each time point. Compared to similar estimators proposed previously in the literature [e.g., [11] this computational strategy considerably reduces the complexity inherent in time-varying network analysis, rendering our approach scalable to high-dimensional datasets with many time points and genes.

## 2 Materials and methods

**Fig 1:**
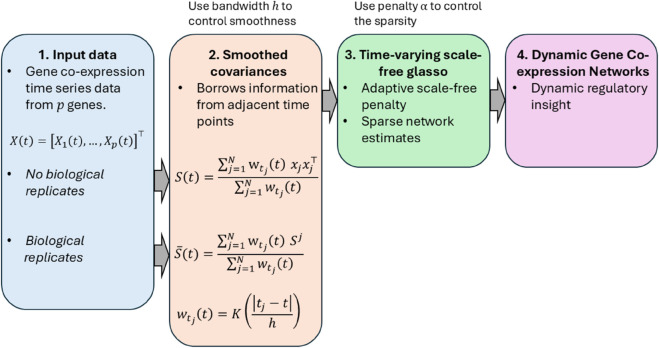
Flowchart of the time-varying scale-free graphical lasso method for dynamic scale-free gene network modeling.

In this section, we will describe the time-varying scale-free network estimation procedure in detail. Steps of our procedure are illustrated in a flowchart in Fig 1.

### 2.1 Time-varying glasso algorithm

Let $A^{⊤}$ be the transpose of a real-valued matrix or vector *A*. Let $X(t)=[X_{1}(t),\ldots ,X_{p}(t){]}^{⊤}$ be a random vector of length *p* following a multivariate Gaussian distribution ${𝒩}_{p}(\mu (t),\Theta^{-1}(t))$ indexed by t∈[0,1], where $\Theta (t)=[\theta_{ij}(t)]$ is the inverse of the time-varying symmetric and positive definite covariance matrix $\Sigma (t)=[\sigma_{ij}(t)]$. We assume that the vectors *X*(*t*) are independent over time *t* and, without loss of generality, that the mean vector μ(t) is zero for all *t*. Moreover, we assume that the precision matrices Θ(t) change smoothly over time. The realization of *X*(*t*) at time *t*_*k*_ is denoted by *x*_*k*_, where k∈I, I={1,…,N}, and $0\leq t_{1}\leq \ldots \leq t_{N}\leq 1$.

Here we assume that the time-varying gene co-expression network can be statistically modeled using GGM. Denote the time-varying GGM by G(t)={V,E(t)}, where V={1,…,p} is the set of nodes (genes) and E(t)={(i,j)∣i≠j} is the set of dynamic edges at time *t*. Edges are determined by the nonzero elements of the precision matrix: (i,j)∈E(t) if and only if $\theta_{ij}(t)\neq 0$. The precision matrix is unknown and is estimated from the observed data *X*(*t*).

Denote the time-invariant p×p sample covariance matrix for *N* observations by $S=\sum_{j=1}^{N}x_{j}x_{j}^{⊤}/N$. Let $\text{tr}(A)=\sum_{i=1}^{p}a_{ii}$ be the trace of a matrix *A*, and let |A| be the determinant. The graphical lasso [8,9] is the estimator of the following minimization problem

$$\hat{\Theta }=\arg\min_{\Theta ≻0}\left\{\text{tr}(\Theta S)-\log|\Theta |+\lambda \|\Theta {\|}_{1}\right\},$$ (1)

where $\left\|\Theta {\|}_{1}=\sum_{i,j=1}^{p}|\theta_{ij}\right|$ is the *L*_1_-norm and *λ* is a positive tuning parameter controlling the sparsity of the estimate $\hat{\Theta }$.

The approach proposed by [12] estimates the precision matrix Θ(t) at time *t* by replacing the sample covariance matrix *S* in (1) with a weighted (smoothed) covariance matrix *S*(*t*), defined as

$$S(t)=\frac{\sum_{j=1}^{N}w_{t_{j}}(t)\;x_{j}x_{j}^{⊤}}{\sum_{j=1}^{N}w_{t_{j}}(t)},$$ (2)

where the weights $w_{t_{j}}(t)$ are determined using a symmetric nonnegative kernel function K(·) with a positive bandwidth parameter *h*

$$w_{t_{j}}(t)=K\left(\frac{\left|t_{j}-t\right|}{h}\right).$$ (3)

#### 2.1.1 Time-varying glasso for biological replicates.

In some applications, multiple realizations of the random vector *X*(*t*) may be available as biological replicates. We use $n_{k}\geq 2$ for all k∈I to denote the number of these replicates at time *t*_*k*_. In this case, we first compute the sample covariance matrix over replications at time *t*_*k*_, denoted by $S^{k}=\sum_{j=1}^{n_{k}}x_{j}x_{j}^{⊤}/n_{k}$. Then we compute the smoothed covariance matrix at time *t*. This special case of a weighted covariance matrix is then defined as

$$\bar{S}(t)=\frac{\sum_{j=1}^{N}w_{t_{j}}(t)\;S^{j}}{\sum_{j=1}^{N}w_{t_{j}}(t)},$$ (4)

with the weights $w_{t_{j}}(t)$ as in (3). Hereafter, we denote by *S*(*t*) the covariance estimator regardless of whether it is computed using estimator (2) or (4), depending on that does the data contain biological replicates.

These weighted covariance estimators leverage information (borrow strength) from adjacent time points, thereby reducing the random error in the precision matrix estimation process. Replacing *S* with *S*(*t*) in the glasso formulation, we write

$${\hat{\Theta }}_{n}(t)=\arg\min_{\Theta ≻0}\left\{\text{tr}(\Theta S(t))-\log|\Theta |+\lambda \|\Theta {\|}_{1}\right\}.$$ (5)

As noted by [12], this minimization problem can be solved using any of the glasso algorithms available in the literature [see, e.g., [19]. We refer to this estimator as the time-varying graphical lasso (tvglasso).

### 2.2 Time-varying glasso for the dynamic scale-free structure of gene networks

We assume that dynamic gene co-expression networks exhibit a scale-free structure, characterized by a few genes with very high connectivity and many genes with low connectivity. To incorporate this structural prior assumption into our estimation process, we replace the uniform tuning parameter *λ* in (5) with an adaptive penalty of the scale-free glasso (sfglasso) proposed by [18]. In this adaptive formulation, the glasso minimization problem becomes

$$\hat{\Theta }(t{)}^{m+1}=\arg\min_{\Theta ≻0}\left\{\text{tr}(\Theta S(t))-\log|\Theta |+\sum_{i,j}\lambda_{ij}|\theta_{ij}|\right\},$$ (6)

where the adaptive penalty parameters are defined as

$$\lambda_{ij}=\left\{ \begin{array}{cc} \alpha \left(\frac{1}{\|\theta (t{)}_{\neg i}^{m}{\|}_{1}+\epsilon_{i}}+\frac{1}{\|\theta (t{)}_{\neg j}^{m}{\|}_{1}+\epsilon_{j}}\right), & \text{if }i\neq j\\ 2\alpha /\epsilon_{i}, & \text{if }i=j. \end{array}\right.$$ (7)

Here, $\left\|\theta (t{)}_{\neg i}^{m}{\|}_{1}=\sum_{j\neq i}|\theta_{ij}^{m}(t)\right|$ denotes the *L*_1_-norm of the *i*th row of $\Theta (t{)}^{m}$ excluding the *i*th component, and $\epsilon_{i}$ are small positive constants ensuring numerical stability. Set $\epsilon_{i}=\theta_{ii}(t{)}^{m}$ as proposed by [18]. In practice, two iterations (one reweighting step, *m* = 2) are typically sufficient [18,20]. We refer to the resulting estimator as the time-varying scale-free graphical lasso (tvsfglasso). We use the identity matrix as the initial value for Θ(t) in the tvsfglasso method.

The proposed tvsfglasso method combines elements from both tvglasso and sfglasso, but offers several important advantages over these and other approaches: First, unlike the model of [12], the inclusion of an adaptive penalty in tvsfglasso makes the estimator particularly suitable for gene co-expression network analysis, as it emphasizes hub structures that are biologically meaningful. Second, compared to the method of [11], tvsfglasso requires substantially less memory and can directly leverage efficient implementations of the glasso, making it more scalable to high-dimensional problems. Third, unlike the sfglasso, which is not applicable to time-series data, tvsfglasso is specifically designed for time-series data. Fourth, we provide software implementation not only for our method (tvsfglasso) but also for scale-free glasso (sfglasso) [18] and the time-varying glasso (tvglasso) [12] [see also [13]. Together, these features make tvsfglasso a novel and practical tool for estimating dynamic gene co-expression networks.

Although [21] have questioned whether gene co-expression networks are truly scale-free, with suggestions that a log-normal distribution might better describe the degree distribution of gene co-expression network. The debate whether real-world networks are scale-free or not is ongoing without a clear conclusion [see, e.g., [22–25]]. The adaptive lasso penalty (7) encourages the appearance of hub nodes. The regularization we are applying can be interpreted as an approximate of a log-normal distribution as noted by [18]. This is in line with the proposal [21] who state that a log-normal distributions with heavier tails might fit the degree distributions as well or even better than power-laws. This makes our estimator a viable option for analyzing real-world gene co-expression time-series data even when the scale-free assumption is not strictly met. Moreover, we have decided to call our estimator as “time-varying scale-free glasso” similar to [18] because the readers might be more familiar with scale-free models [see, e.g., the widely applied method proposed by [26].

Because the smoothing of time points is done before solving the glasso problem, the computational complexity of the tvsfglasso estimator is directly proportional to the complexity of the underlying glasso algorithm and the complexity of corresponding software implementation. This greatly reduces the memory usage compared to methods like [11]. Specifically, the tvsfglasso requires running the glasso algorithm *m* times at each time step (in practice, two iterations are typically sufficient). Multiple fast algorithms and software implementations exist for solving the glasso minimization problem, including those described in [8,19,27,28].

The computational time of our method further scales linearly with the number of time steps in the time series. To the best of our knowledge, this represents the most time-efficient strategy currently available for estimating dynamic gene co-expression networks from time-series data using GGMs. For example, using our tvsfglasso method, it takes approximately 168 seconds to analyze a time series with 200 time points, 10 replications, and 500 variables when the algorithm and software of [28] are run on a standard desktop computer for 25 different values of *α* and *m* = 2.

We remind that the selection of the optimal bandwidth parameter *h* in (3) is a challenging research question: some smaller changes in the gene co-expression topology happening during a narrow time window might be observed only with small values of *h*, whereas larger changes spanning over a wider time window are observed with larger values of *h*. For now, we recommend users of our method and the method proposed by [12] to utilize several different values of the bandwidth parameter *h* instead of single value. This type of approach is commonly used in scale-space analysis [see, e.g., [29–31]].

In the following sections, we demonstrate that the tvsfglasso yields networks that are biologically more meaningful when the underlying sparse graphical model exhibits a dynamic scale-free structure.

## 3 Results

### 3.1 Simulation analysis

Simulation examples are useful in statistical analysis because they test methods under controlled conditions with a known ground truth. Even then, they highlight how data variability affects performance and reveal the methods’ limitations. Therefore, we compare different estimators with our tvsfglasso and tvglasso approaches using covariance smoothing (4). In particular, we compare tvglasso and tvsfglasso with estimators that use the time-invariant sample covariance matrix instead of estimators (2) or (4). These estimators are: (i) glasso when the whole times-series data set is first used to estimate a single sample covariance matrix *S*. We call this estimator as pooled glasso (pglasso); (ii) glasso when the sample covariance matrix is estimated at each time point (without smoothing); and (iii) the time-invariant scale-free glasso (sfglasso) [18] when the covariance matrix is estimated at each time point (without smoothing). We use the R package glassoFast [28], which implements an efficient algorithm to solve the glasso minimization problem (6). We set *p* = 100 and the number of replicates *n*_*k*_ as 10, 50, and 100 for each time point.

Assumption that the nodes (genes) follow a preferential attachment mechanisms over time implies some specific restrictions to the network simulation model. In our simulations, we make the following assumptions:

1. Relatively few edges are deleted and added at each time point.
2. The degree distribution of the sparse dynamic network remains scale-free over time.
3. The graph remains acyclic over time.
4. Nodes with lower degrees have a higher probability of experiencing changes in their connected edges over time.

Our simulation procedure is described in detail in S1 Text.

To comprehensively evaluate our method, we employed three distinct scale-free network simulators. To initialize our dynamic scale-free simulator, we used the following network generators: (i) the scale-free network constructed using the algorithm of [32] available in the R package huge; (ii) the scale-free network constructed using the Liu and Ihler algorithm [18]; and (iii) a smooth scale-free network combined with the algorithm of [12]. All of these simulators produce scale-free networks using the Barabási–Albert (BA) preferential attachment model, a commonly used method for generating scale-free structures. They also include their own procedures for generating the corresponding precision matrices. We initialize the preferential attachment model of the simulators by [32] and [18] with two nodes and the simulators by [12] with four nodes.

In addition to scale-free networks, we considered three non-scale free network models: (i) the super-hub network constructed using the algorithm of [33]; (ii) “rapidly changing” network constructed using the Yang and Peng algorithm [13]; and (iii) an Erdős-Rényi network available in the R package huge. We chose to include them for specific reasons. First, the model by [33] introduces so-called “super-hubs” (nodes with exceptionally high degree) which results in a network with a heavy-tailed degree distribution resembling that of real-world gene co-expression networks. Second, the model by [13] and the Erdős-Rényi model are used to assess the generalizability and robustness of the tvsfglasso estimator, in other words, to assess whether the tvsfglasso can be used to capture the structure of the dynamic network even when the scale-free assumption is incorrect, without being forced into systematically incorrect estimates.

The tvsfglasso has two tuning parameter values: i) The bandwidth parameter *h*; and ii) the tuning parameter *α*. In these simulations, we use bandwidth parameter values h∈{0.01,0.5,1,1.5,2.5} to see how robust tvsfglasso is with respect to different values of *h*. Moreover, we use Epanechnikov kernel function in (3), that is K(a)=3/4(1 − *a*)^2^ for |a|≤1 similar to [13]. We select the tuning parameter *α* from 25 candidate values between 0.05 and 0.3 on a logarithmic scale. In these simulations, we use an extended Bayesian Information Criterion (eBIC) [34] variant to choose the graphical model at each time step. In particular, our eBIC variant is

$$eBIC_{\gamma }[\hat{\Theta }(t_{k})]=-Nl[\hat{\Theta }(t_{k}),S(t_{k})]+\log(N)df+4\gamma \log(p)df,$$ (8)

where $l[\hat{\Theta }(t_{k}),S(t_{k})]=\log(|\hat{\Theta }(t_{k})|)$ − $\text{tr}[S(t_{k})\hat{\Theta }(t_{k})]$. Parameter *df* is the size of the edge set of a candidate graph. Parameter *γ* is a positive eBIC specific tuning parameter that controls the model complexity. Large values of *γ* will yield sparse graphical models. When using stationary and pooled glasso and sfglasso, we use the eBIC proposed by [34] to select the tuning parameter *λ* among 25 candidate values.

**Fig 2:**
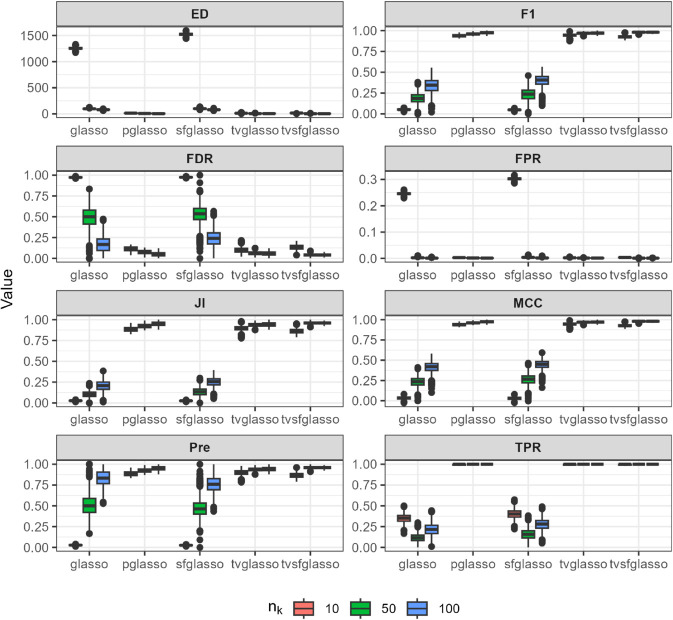
Analysis of data replicates generated by the R package huge [32] (a scale-free model) using different glasso variants and tvsfglasso at h=1. Different panels represent different binary classification metrics.

**Fig 3:**
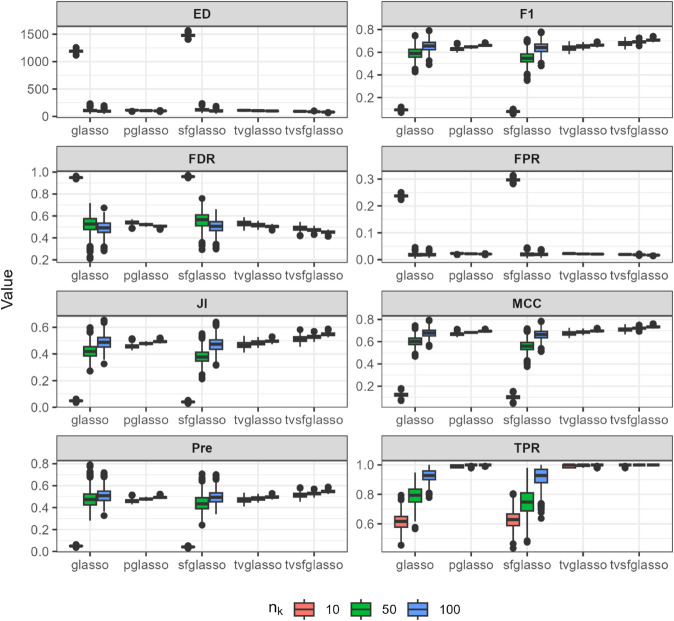
Analysis of data replicates generated by simulator described in [18] (a scale-free model) using different glasso variants and tvsfglasso at h=1. Different panels represent different binary classification metrics.

**Fig 4:**
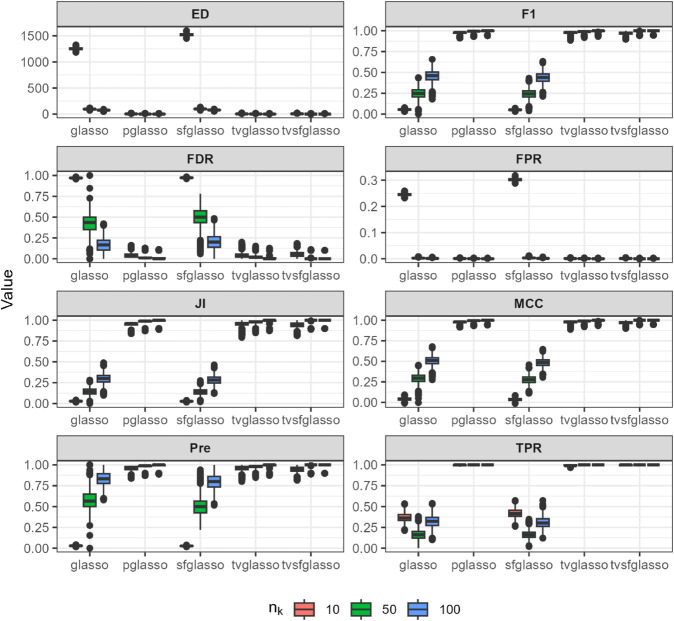
Analysis of data replicates generated by simulator described in [12] (a smooth scale-free model) using different glasso variants and tvglasso and tvsfglasso at h=1. Different panels represent different binary classification metrics.

**Fig 5:**
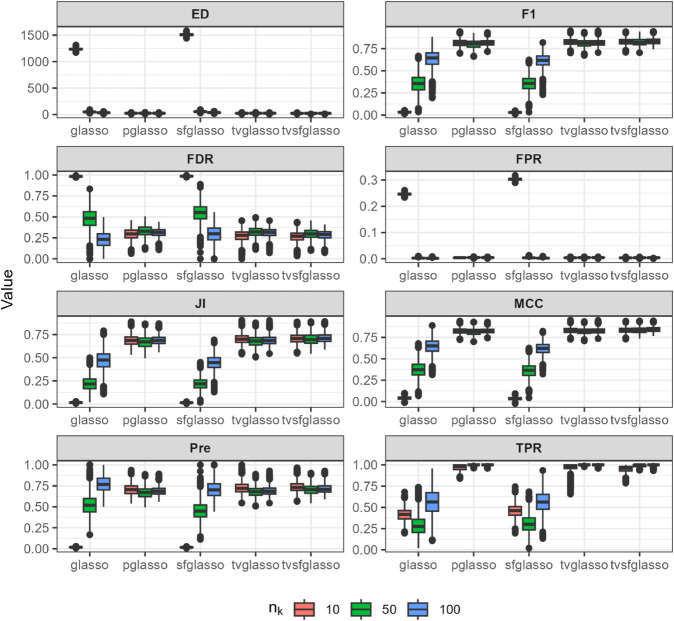
Analysis of data replicates generated by simulator described in [33] (a super-hub model) using different glasso variants and tvglasso and tvsfglasso at h=1. Different panels represent different binary classification metrics.

**Fig 6:**
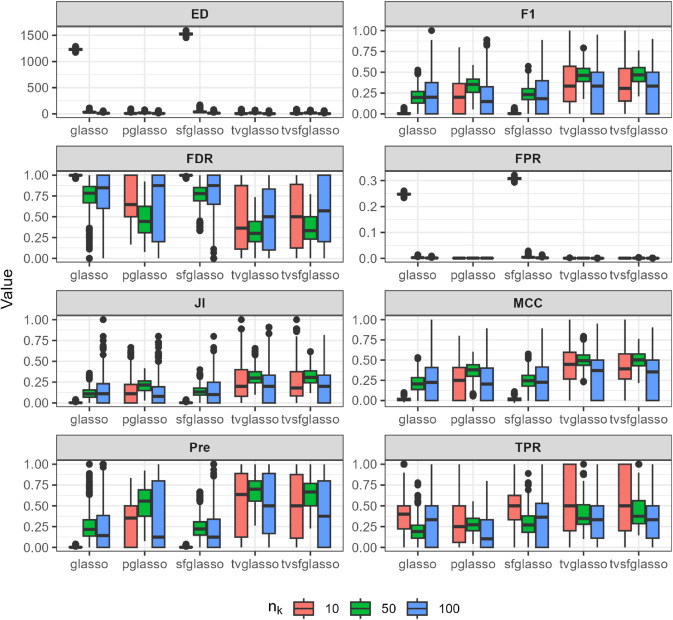
Analysis of data replicates generated by simulator described in [13] (non-scale-free network) using different glasso variants and tvglasso and tvsfglasso at h=1. Different panels represent different binary classification metrics.

**Fig 7:**
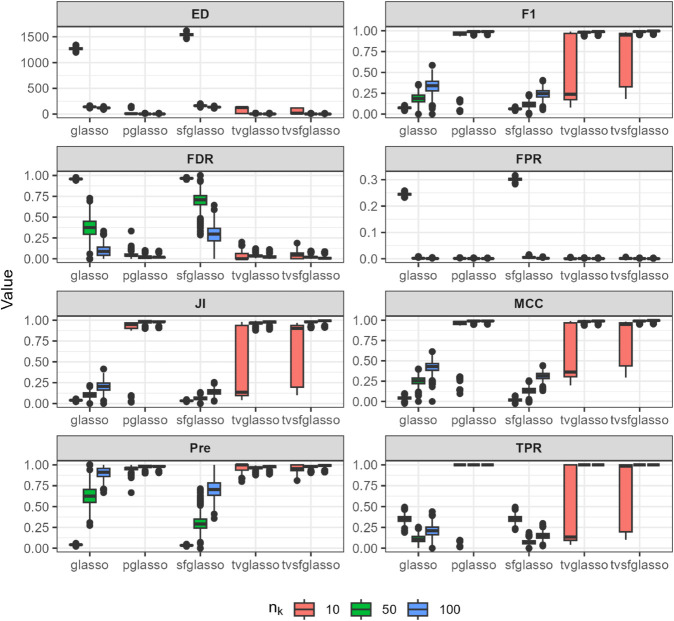
Analysis of data replicates generated by simulator described in [12] (smooth Erdős-Rényi graph) using different glasso variants and tvglasso and tvsfglasso at h=1. Different panels represent different binary classification metrics.

We evaluate the quality of network estimates produced by glasso, pglasso, sfglasso, tvglasso, and tvsfglasso on simulated time-series data using eight binary classification and network structure-oriented evaluation metrics: F1 score, false discovery rate (FDR), false positive rate (FPR), Matthews correlation coefficient (MCC), precision (Pre), true positive rate (TPR), Jaccard index (JI), and graph edit distance (ED). Definitions of these metrics are provided in S1 Text. We repeat the simulation procedure 10 times to reduce the effect of random sampling and the averaged simulation results are illustrated in Figs 2–7 at *h* = 1 as proposed in [12] and γ=0.5 as proposed in [34].

As shown in Figs 2, 3, and 4, when the underlying dynamic graphical model is indeed a scale-free network undergoing minor changes over time, the tvglasso and tvsfglasso estimators improve estimation accuracy remarkably compared to the stationary glasso and sfglasso. For example, the True Positive Rate (TPR) (and the Edge Distance (ED)) associated with tvsfglasso is much higher (lower) when *n*_*k*_ = 10 compared to that of the stationary glasso and sfglasso even when *n*_*k*_ = 100. Moreover, the additional structural information incorporated by tvsfglasso yields slightly higher averaged Precision, JI and MCC values and lower FDR, and ED values than tvglasso. For detailed comparisons between tvglasso and tvsfglasso, see Figs B–E in S1 Text.

It seems that tvglasso and tvsfglasso return reasonable estimates of the dynamic graphical model when the bandwidth parameter is not too small. Only when *h* is extremely small (*h* = 0.01), the binary classification metrics suggest that the smoothing is inadequate and network estimates are computed without sharing information from the adjacent time points. For more details, see Figs G–L in S1 Text.

The pglasso estimator appears to yield reasonable estimates in these examples. However, its accuracy largely reflects the small changes in the dynamic networks and corresponding precision matrices, as well as the fact that the data are generated independently at each time step. Consequently, although a consensus network is quite similar to ones obtained at each time point, the pglasso estimator cannot be used to inspect structural changes over time. Furthermore, when the networks are simulated using the scale-free procedures, the tvsfglasso achieves much higher averaged F1, JI, Pre, and MCC values and lower FDR, FPR, and ED values compared to pglasso (see Figs A and C in S1 Text).

In contrast, when the underlying network is not scale-free or its topology changes abruptly, there is a clear drop in estimation accuracy. In Fig 5, both tvglasso and tvsfglasso still yield better averaged binary classification metrics compared to their stationary counterparts. However, this improvement is primarily due to the smoothed covariance matrix estimator, as the difference between tvglasso and tvsfglasso becomes negligible.

When examining the non-scale free data constructed using the model proposed by [13] (Fig 6), tvsfglasso does not improve the estimation accuracy. This limitation is observed with all the estimators under these conditions; thus, based on these simulation results, none of the methods considered are adequate for this specific network inference problem. We note that the estimators (5) and (6) are designed for smoothly changing networks. In line with this limitation, tvsfglasso is not designed to analyze network with abrupt topological changes or any type of networks/degree distributions.

In Fig 7, it can be seen that as the sample size increases, both tvglasso and tvsfglasso accurately recover the underlying dynamic graphical model. Although the adaptive lasso penalty in tvsfglasso promotes the emergence of hub nodes in smoothly evolving gene networks, the differences between tvglasso and tvsfglasso in terms of ED, JI, F1, and MCC are negligible. When *n*_*k*_ = 10, tvsfglasso tends to yield slightly higher MCC, JI, and F1 values on average; however, the variability of these metrics remains substantial for both methods, suggesting that larger sample sizes are needed for reliable network estimation (see Fig F in S1 Text). Nevertheless, it seems that even when the underlying network does not exhibit high-degree nodes, tvsfglasso produces network structures consistent with those obtained by tvglasso, indicating that the method does not impose an incorrect structural bias.

Authors in [34] noted that although small values of eBIC parameter *γ* tend to yield higher positive selection rates, whereas larger values of *γ* are associated with lower false discovery rates, this trade-off diminishes as both *n* and *p* grow large. To examine whether this property holds for our eBIC variant, we conducted a small sensitivity analysis with different configurations of the parameter *γ* at n=10,50,100 when analyzing the scale-free model of [18]. See Figs M, N, and O in S1 Text. The results indicate that the eBIC variant we employed is a relatively robust model selection tool, showing no serious overfitting or underfitting issues across the tested values of *γ*. Moreover, reasonable estimates are obtained across a range of *γ* values as the sample size increases, γ=0.5 being a reasonable rule-of-thumb value. These findings are consistent with the original model selection criterion proposed by [34].

Note that we have used eBIC as a preliminary model selection criterion to demonstrate the usage of our estimator. Arguably, a general model selection criteria for dynamic GGMs is still lacking in the research literature.

While scale-free network modeling is commonly used to capture the hub-like structure of gene networks arising from preferential attachment, gene co-expression networks may also exhibit other dynamic features, such as changes in community or hierarchical structure, over time. This limitation of the sfglasso estimator is noted by [18]. Nevertheless, the primary focus of this paper is to propose a scalable and time-efficient estimator designed to capture high degree genes (hub genes) which presence might be explained by a preferential attachment mechanism among the genes. We also remind that because there is no such thing as a free lunch in statistics, a researcher seeking a comprehensive analysis of gene co-expression networks should always consider a diverse set of network estimators and analytical tools.

### 3.2 Dynamic *Drosophila melanogaster* co-expression network

We applied tvsfglasso on a public gene expression time-series data of *Drosophila melanogaster* (fruitfly) available at https://www.ncbi.nlm.nih.gov/geo/query/acc.cgi?acc=GSE121160 [35], see also [36–38]. The data contains gene expressions collected from the whole embryos of *Drosophila melanogaster* measured at 14 time points during the first 20 hours of development (0, 1, ..., 6, 8, 10, ..., 20 hours). Moreover, each sample was measured in biological quadruplicates (*n*_*k*_ = 4 for all k=1,…,14). Thus, we used the smoothed covariance matrix (4) together with the tvsfglasso. We focus our analysis to the 996 Transcription Factors (TFs) listed by [36]. From these TFs, we chose such genes that had observed non-zero counts in at least 20 samples and more than 10 counts on average. After this, we $\log_{2}$ transformed the data, $y=\log_{2}(x+1)$ where *x* is the original count and *y* is the $\log_{2}$ transformed count. Finally, we normalized the data to have mean zero and standard deviation one. Not to miss too many biologically important but stably expressed TSs while still alleviating the high-dimensionality of the problem, we chose subset of 400 TFs with the highest variance.

**Fig 8:**
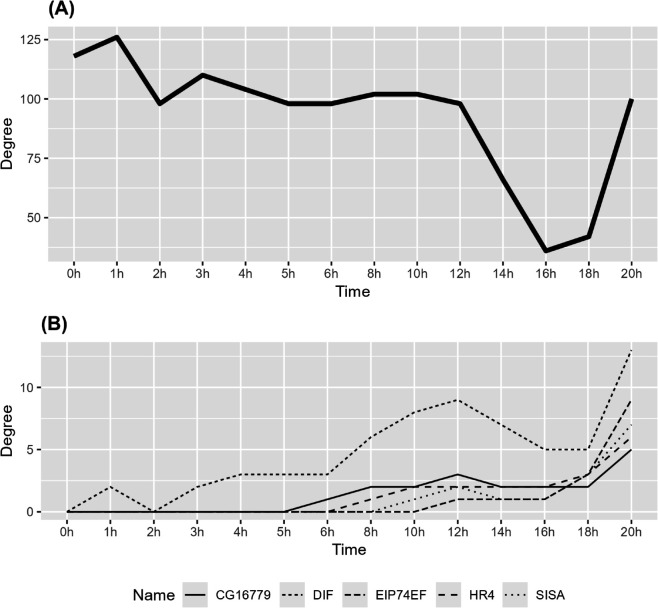
Estimated degree of the time-varying TFs network constructed using tvsfglasso at h=1 (A) for the whole network and (B) for the TFs with the greatest increases in degree, as determined by the highest slope coefficients $\beta_{1}^{i}$, ${degree}_{TF}^{i}=\beta_{0}^{i}$  +  $\beta_{1}^{i}$ time. Different line types represent different TFs.

When computing the time-varying TF networks, we set the bandwidth parameter *h* = 1 and used the Epanechnikov kernel function. We selected the tuning parameter *α* of the tvsfglasso estimator using the same eBIC variant as in the simulation examples, choosing from 50 candidate values between 0.05 and 0.15 on a logarithmic scale at γ=0.5. The degree of the dynamic TF network and specific nodes are illustrated in Fig 8. The TF networks estimated at each time step and the difference graphs between consecutive time steps are illustrated in Figs P–AB in S1 Text.

**Fig 9:**
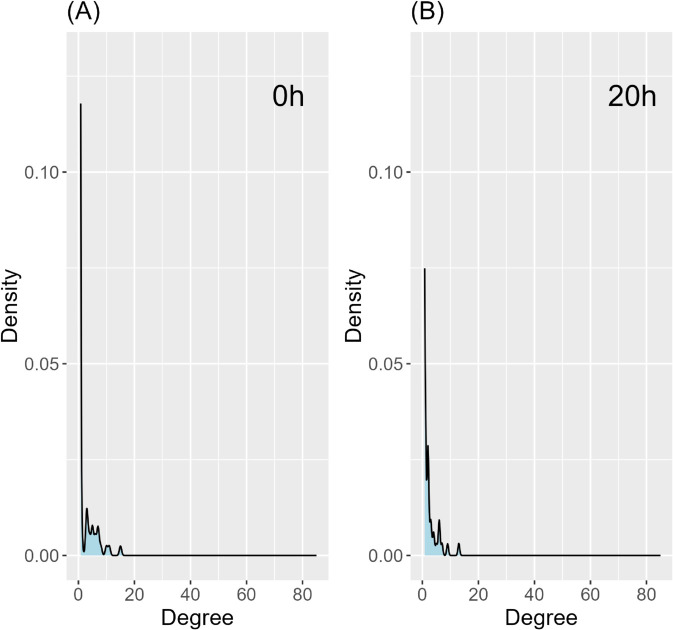
Density estimates of degree distributions of the time-varying *Drosophila melanogaster* TF networks constructed with tvsfglasso at h=1 at time-points (A) 0h, and (B) 20h.

Fig 8(**A**) shows that how the degree of the *Drosophila melanogaster* TF network changes over the 20-hour period. We identified the five TFs with the largest degree increases for more detailed degree analysis by selecting those with the highest slope coefficients $\beta_{1}^{i}$ (i=1,…,400) from the linear regression ${degree}_{TF}^{i}=\beta_{0}^{i}+\beta_{1}^{i}\;\text{time}$. The degree profiles of these TFs are shown in Fig 8(**B**). The tvsfglasso analysis indicates that these TFs become more active prior to the larva stage and the degree of these TFs is higher at 20 hours compared to the initial stage. In Fig 9, we illustrate the degree distribution of the networks constructed using tvsfglasso at time points 0 hours and 20 hours. The density estimates show that the degree distribution of the TF networks constructed using tvsfglasso exhibits a right-skewed (power-law type) distribution, which is commonly observed in biological networks.

**Fig 10:**
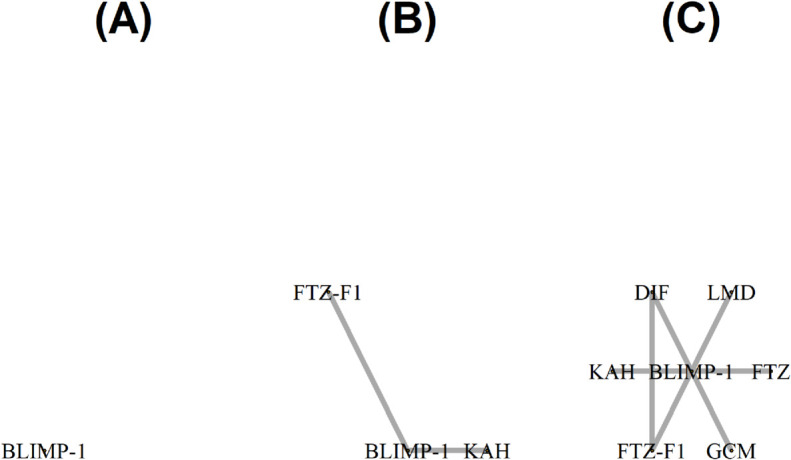
The neighborhood of BLIMP-1 in the *Drosophila melanogaster* networks at times-points (A) 16h, (B) 18h, and (C) 20h when the network is constructed using tvsfglasso at h=1.

For more detailed analysis, we also examined the neighborhood of TFs BLIMP-1, TFZ-F1, and FTZ. BLIMP-1 is a transcriptional repressor known to silence FTZ-F1 (or delays its expression) at the initiation of metamorphosis [see, e.g., [39]. Fig 10 illustrates how the neighborhood of BLIMP-1 at the final three time points change over time. The analysis of the dynamic networks constructed with tvsfglasso identifies an interaction between TFs BLIMP-1 and FTZ-F1 (FTZ) at approximately 18 hours (20 hours), just before the transition from the embryonic to the larval stage. This is also in line with the previous studies that FTZ often works in co-regulated way with FTZ-F1; FTZ-F1 may act as a co-factor for FTZ binding in some enhancers [see, e.g., [40].

Figs 8, 10, and Figs P–AB in S1 Text show an overall drop and sudden increase in significant TF-TF connections over the 0 – 20 h time interval. From Fig 8 and Figs V, Y–AA in S1 Text we can see two prominent topological shifts: one between 12 – 16 h, and another between 16 – 20 h. The first change might happen because repressors such as BLIMP-1 which we inspected in more detail become active, silencing some other TFs. The later shift occurs just before the embryo-to-larva transition in *Drosophila melanogaster*, coinciding with major developmental changes. A similar observation in network density was previously reported in a longer, but more irregularly sampled, time-series analysis of the *Drosophila melanogaster* co-expression network [41].

We also analyzed the data using tvglasso and selected the network models using the eBIC. However, in this analysis, the resulting networks were graphical models with no edges (i.e., empty graph). This limitation is likely due to the smaller sample size compared to the simulation examples (4 × 14 samples vs. 10 × 200 samples). Nevertheless, this finding highlights how incorporating structural information via tvsfglasso can alleviate the challenges typical in high-dimensional co-expression network analysis whereas the time-varying glasso variants with fusion penalty [see, e.g., [11] cannot be applied to large scale high-dimensional problems.

## 4 Discussion

We have shown that the tvsfglasso estimator is well-suited for time-varying gene co-expression network analysis when the underlying network topology changes over time and exhibits a scale-free structure. The limitation of tvsfglasso is that it tries to capture the dynamic nature of the network by applying kernel smoothing to the sample covariance matrices prior to precision matrix (network) estimation. In contrast to tvsfglasso, the method proposed by [11] enforces smoothness across time-points through a fusion penalty, promoting similarity in the precision matrix estimates. Our design choice is a deliberate trade-off: By avoiding explicit fusion penalties, tvsfglasso remains time-efficient, memory-light, and directly compatible with fast glasso algorithms, making it a feasible tool in high-dimensional setting. Thus, tvsfglasso is best suited for applications where computational efficiency and interpretability are needed. Other time-varying glasso variants with fusion penalty [see, e.g., [11] remain more appropriate when explicit control over network evolution smoothness is required. Arguably, a fast algorithm and software implementation for these kind of minimization problems is still lacking in the research literature.

There is no consensus on how to simulate dynamic GGMs, and countless parameter choices can yield vastly different network behaviors and time-series data. To demonstrate this, we employed multiple simulators, each capturing a different aspect of scale-free networks, such as preferential attachment (hub-dominated topology), super-hubs, and abrupt topological shifts. These simulation examples show that when the topology of a scale-free network does not undergo abrupt changes, tvsfglasso returns dynamic network estimates with low error and high statistical power.

For future studies, we plan to apply tvsfglasso to an infant microbiome analysis. In this context, we aim to examine how microbial relationships evolve using data collected during the early months of life from several newborns [see, e.g., [42]. See [43] for a review.

A more challenging problem than developing a time-efficient algorithm for the time-varying graphical lasso is the issue of model selection. For instance, [11] proposed using cross-validation for model selection, but this approach requires the user to predefine an appropriate penalty function, assuming prior knowledge about how the underlying network evolves over time. If we were interested exclusively in the hub nodes of the time-varying co-expression network, we could apply the hub-detection procedure proposed by [31] with tvsfglasso at each time step.

The choice of a suitable model selection criterion plays a critical role in the final outcome and substantially influences the interpretation of the resulting networks. While tvsfglasso integrates additional structural information into the gene co-expression network analysis, employing a model selection criterion that reflects the essential properties of gene co-expression networks could further enhance the reliability of time-varying network estimation. Identifying such a criterion is left for future research.

Although the validity of scale-free network models is debatable, they are still commonly used to capture the hub-like structure of gene networks [26]. While this paper focuses on preferential attachment models, gene co-expression networks may also exhibit other dynamic features, such as changes in community or hierarchical structure over time. This limitation of the sfglasso estimator has been noted by the authors of the sfglasso [18]. Nevertheless, tvsfglasso introduced in this paper is a scalable and time-efficient estimator designed to examine the dynamic preferential attachment mechanism among genes using high-dimensional time-series data assuming that a power-law model can be used to describe the degree distribution of a gene co-expression network. For researchers seeking a comprehensive analysis of gene co-expression networks, a diverse set of network estimators and analytical tools is recommended.

## Supporting information

S1 Text Includes all supporting information materials. (PDF)
