# Supplementary material for: tvsfglasso: Time-varying scale-free graphical lasso for network estimation from time-series data
Source: PLoS Comput Biol. 2025 Nov 17;21(11):e1013710. doi: 10.1371/journal.pcbi.1013710 (PMC12633906; doi:10.1371/journal.pcbi.1013710)
Supplement: S1 Text — (PDF) [file pcbi.1013710.s001.pdf]

# tvsfglasso: time-varying scale-free graphical lasso for network estimation from time-series data - Supplementary materials

Markku Kuismin and Mikko J. Sillanpää

Research Unit of Mathematical Sciences
University of Oulu
markku.kuismin@oulu.fi

## Simulation examples - binary classification metrics

Let $\hat{G}(t)$ denote the estimated graph and $G(t)$ the true graph at time $t$. We define

- $TP = \big|\{(i,j) \colon (i,j) \in \hat{G}(t) \text{ and } (i,j) \in G(t)\}\big|$ = the number of edges correctly recovered (the number of true positives).
- $FP = \big|\{(i,j) \colon (i,j) \in \hat{G}(t) \text{ and } (i,j) \notin G(t)\}\big|$ = the number of spurious edges (the number of false positives).
- $TN = \big|\{(i,j) \colon (i,j) \notin \hat{G}(t) \text{ and } (i,j) \notin G(t)\}\big|$ = the number of correctly omitted non-edges (the number of true negatives).
- $FN = \big|\{(i,j) \colon (i,j) \notin \hat{G}(t) \text{ and } (i,j) \in G(t)\}\big|$ = the number of missed true edges (the number of false negatives).

F1 score, precision (Pre), true positive rate (TPR), false positive rate (FPR), false discovery rate (FDR), Matthews correlation coefficient (MCC), Jaccard index (JI), and graph edit distance (ED) are defined as follows,

- $F1 = (2TP)/(2TP + FP + FN)$
- $Pre = TP/(TP + FP)$
- $TPR = TP/(TP + FN)$
- $FPR = FP/(FP + TN)$
- $FDR = FP/(FP + TP)$
- $MCC = \frac{TP \times TN - FP \times FN}{\sqrt{(TP+FP)\times(TP+FN)\times(TN+FP)\times(TN+FN)}}$
- $JI = TP/(TP + FN + FP)$
- $ED = FP + FN.$

If $\sqrt{(TP + FP) \times (TP + FN) \times (TN + FP) \times (TN + FN)} = 0$, we set $MCC = 0$.

## Simulation examples - simulation procedure

We compute edge betweenness centrality to identify edges connected to both low- and high-degree nodes. We denote the number of shortest $(i, j)$-paths by $\sigma(i, j)$, and use $\sigma(i, j|e)$ to denote the number of those paths passing through edge $e$. The edge betweenness centrality of edge $e$ $EB(e)$ is defined as

$$EB(e) = \sum_{i,j \in V} \frac{\sigma(i, j|e)}{\sigma(i, j)}, \tag{1}$$

where $V$ is the set of nodes. Betweenness centrality $EB(e)$ is the sum of the fraction of all-pairs shortest paths that pass through an edge $e$ [see, e.g., 2]. Finally, $EB(e)$ values are normalized by the inverse of the maximum number of vertices, that is $2/(p(p-1))$.

Our simulation procedure can be described as follows:

1. Initialize the process by simulating a network and setting it as $G(0)$. A scale-free type network can be simulated using the Barabási–Albert (BA) preferential attachment model [1].

2. Compute the $EB(e)$ of each edge. Denote these with $EB(e_q)$, $i = 1, \ldots, Q$ where $Q$ is the number of edges in the network at time $t = 0$.

3. At each time step $t_k$, $k = 1, \ldots, N$ remove the edge $e_q$ with probability $P(EB(e_q) < U)$, where $U \sim Unif(a, \max\{EB(e_q)\})$ from the network $G(t_k)$. User defined parameter $a > 0$ controls the sparsity of the scale-free network.

This procedure ensures that, i) the dynamic network is scale-free at each time step, and; ii) edges connected to low-degree nodes are removed with high probability, while edges linked to high-degree nodes (hubs) remain relatively stable.

# Simulation results

Simulation results of the main paper. Supplementary Figures B, A, C, D, and E illustrate the averaged results (over replicates) for pooled glasso (pglasso), time-varying glasso (tvglasso) at $h = 1$, and time-varying scale-free glasso (tvsfglasso) at $h = 1$, $p = 100$.

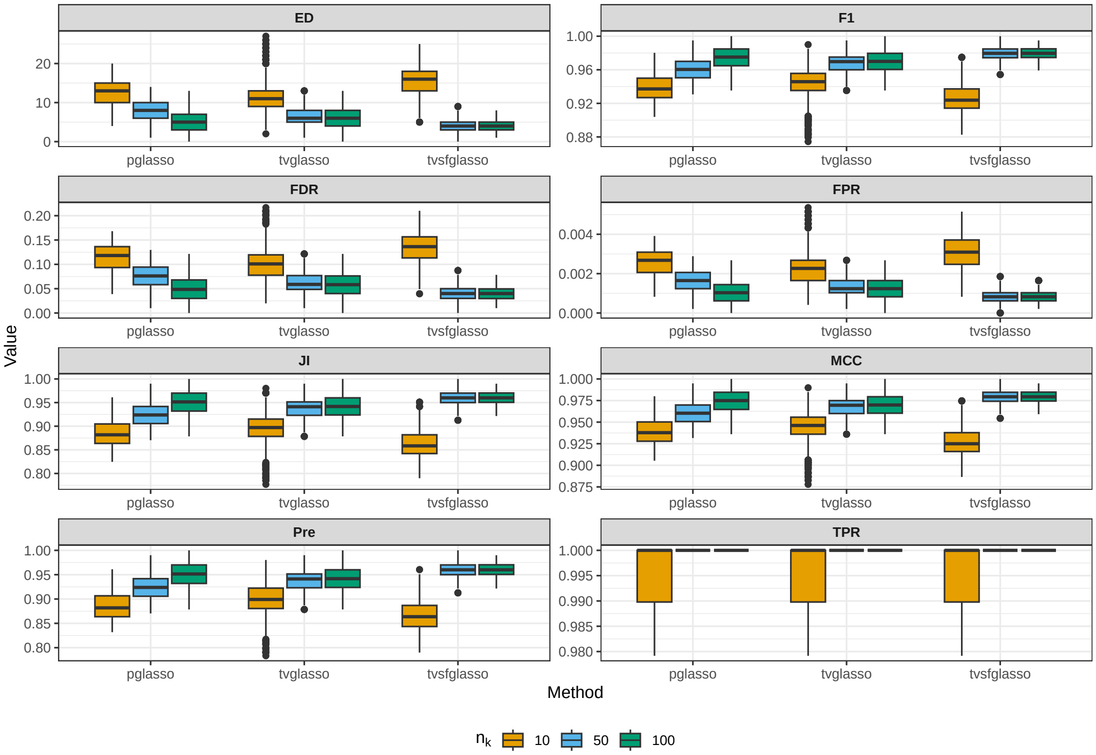


Fig A: Simulator adapted from the R package `huge` [6] (a dynamic scale-free network) using different glasso variants. Different panels represent different binary classification metrics.

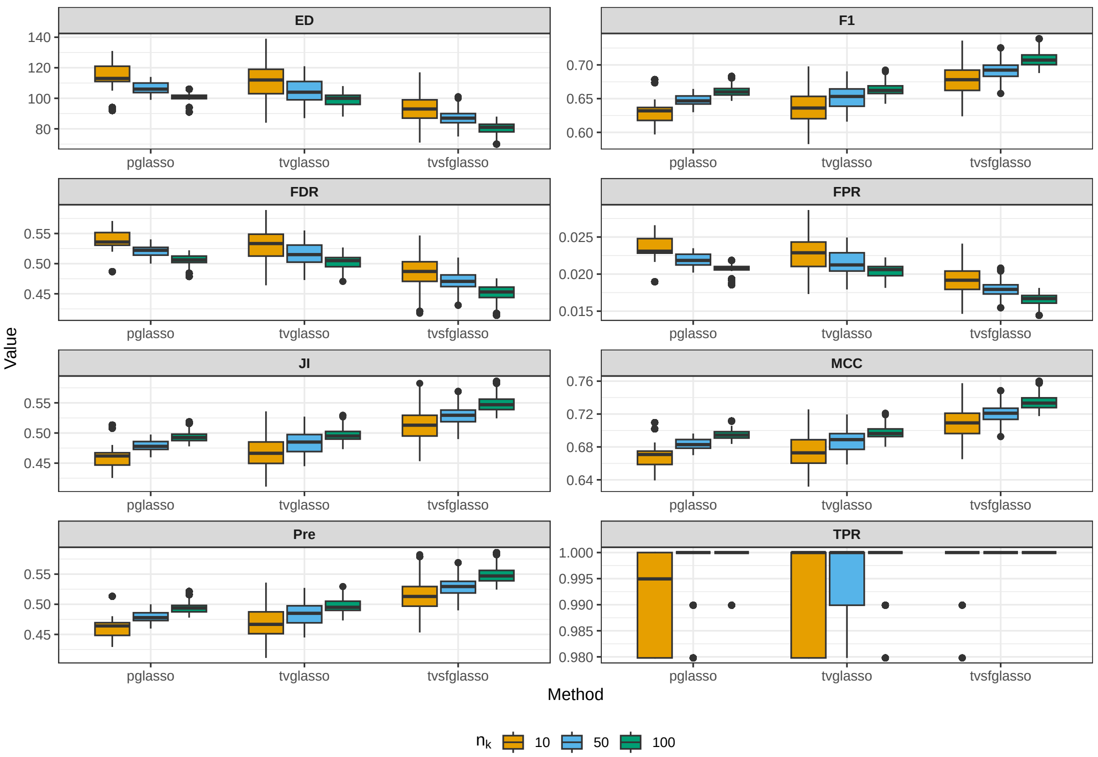


Fig B: Analysis of replicated data sets generated by simulator described in [3] (a dynamic scale-free network) using different glasso variants. Different panels represent different binary classification metrics.

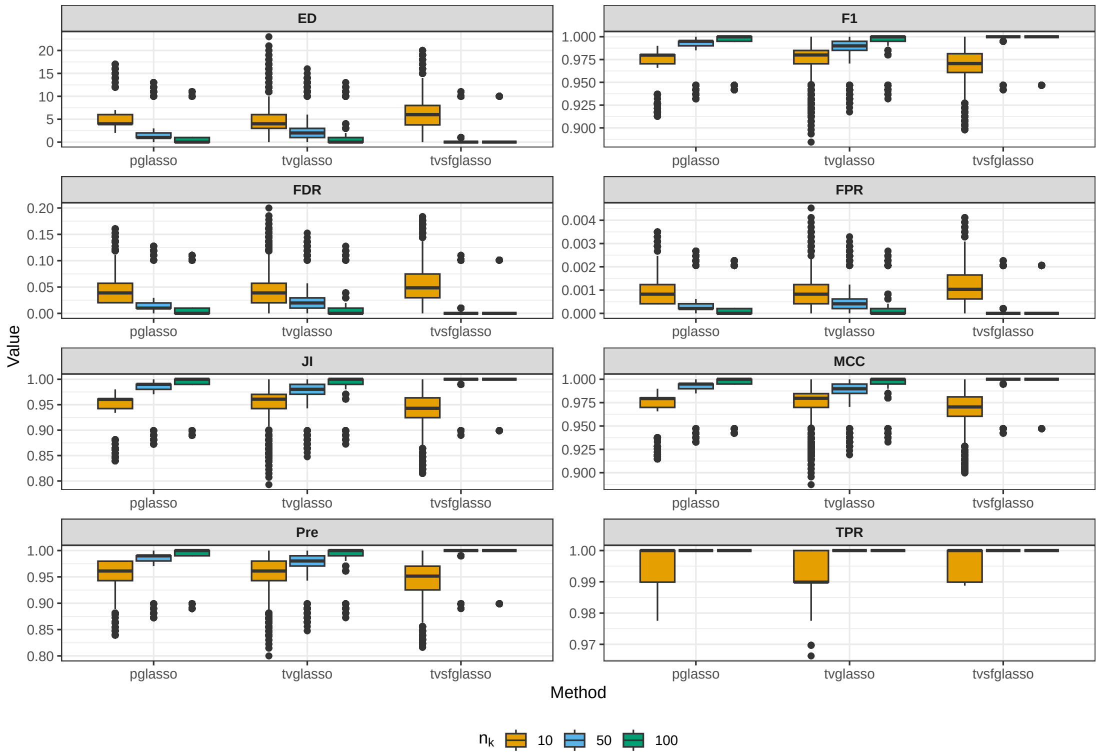


Fig C: Analysis of replicated data sets generated by simulator described in [7] (a smooth dynamic scale-free network) using different glasso variants. Different panels represent different binary classification metrics.

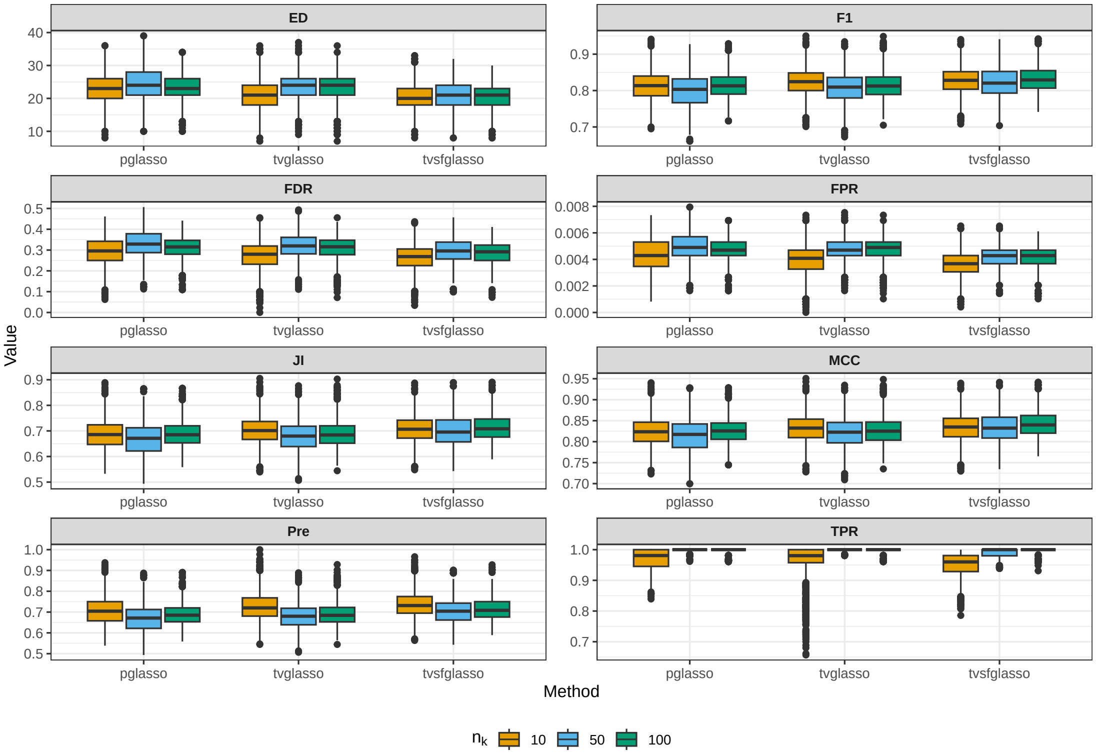


Fig D: Analysis of replicated data sets generated by simulator described in [4] (a dynamic super-hub model) using different glasso variants. Different panels represent different binary classification metrics.

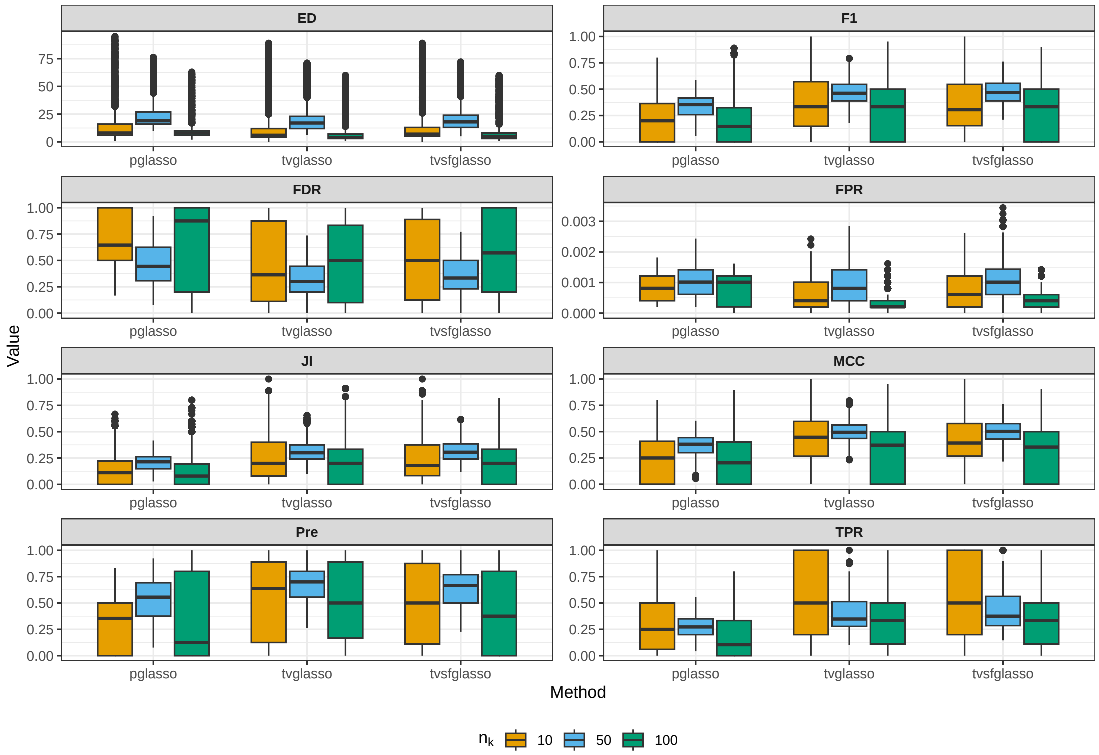


Fig E: Analysis of replicated data sets generated by simulator described in [5] (a dynamic non-scale-free network) using different glasso variants. Different panels represent different binary classification metrics.

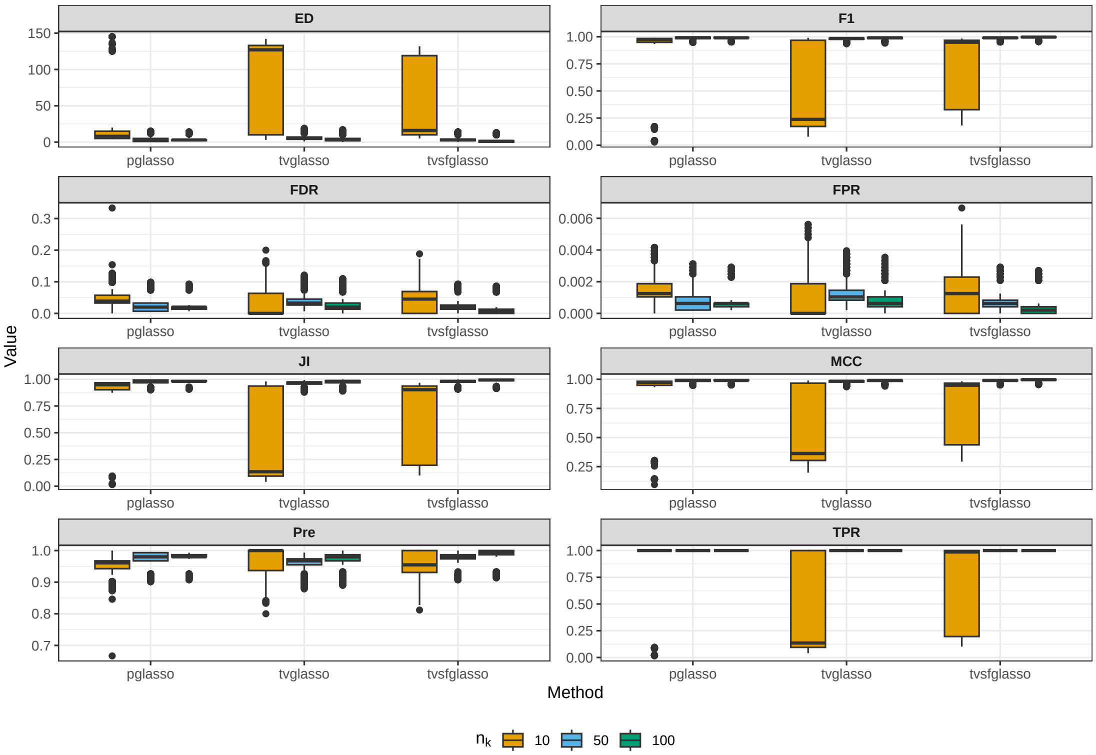


Fig F: Analysis of replicated data sets generated by simulator described in [7] (smooth Erdős-Rényi graph) using different glasso variants. Different panels represent different binary classification metrics.

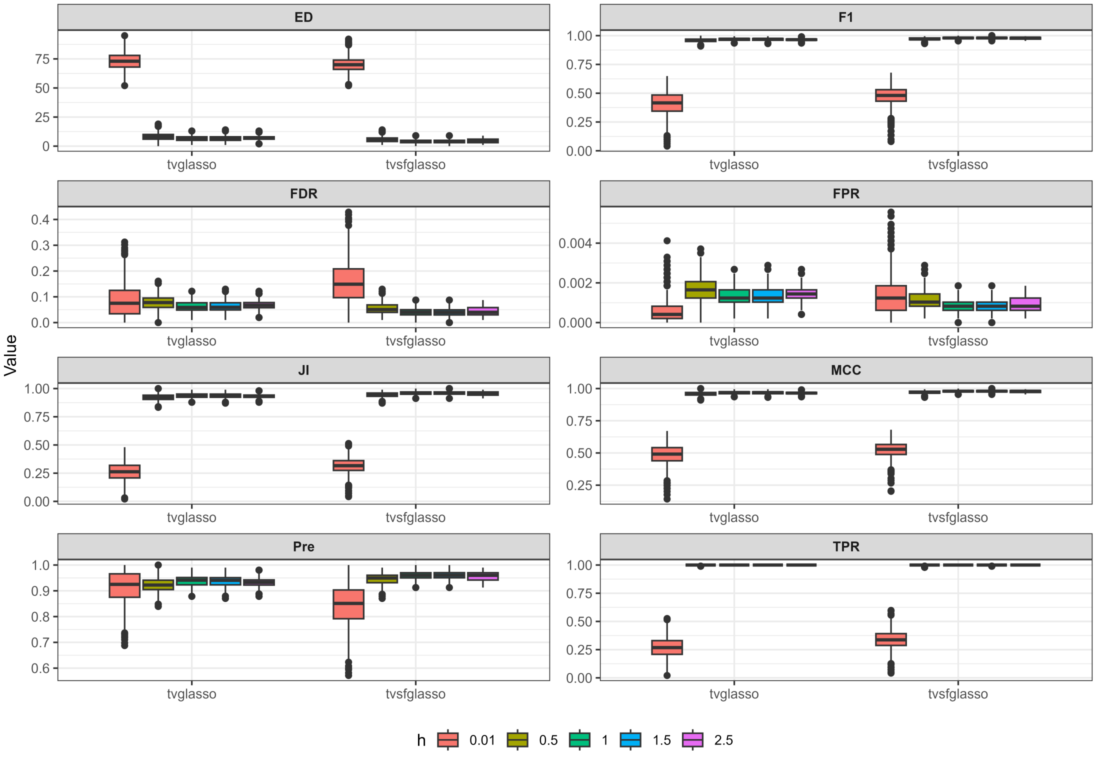


Fig G: Simulator adapted from the R package `huge` [6] using tvglasso and tvsfglasso at different values of the bandwidth parameter $h$. Different panels represent different binary classification metrics when $n = 50$.

# Simulation results - bandwidth

Simulation results of the main paper. Supplementary Figures H, G, I, J, and K illustrate the averaged results (over replicates) for time-varying glasso (tvglasso), and time-varying scale-free glasso (tvsfglasso) at $h \in \{0.01, 0.05, 1, 1.5, 2.5\}$, $p = 100$.

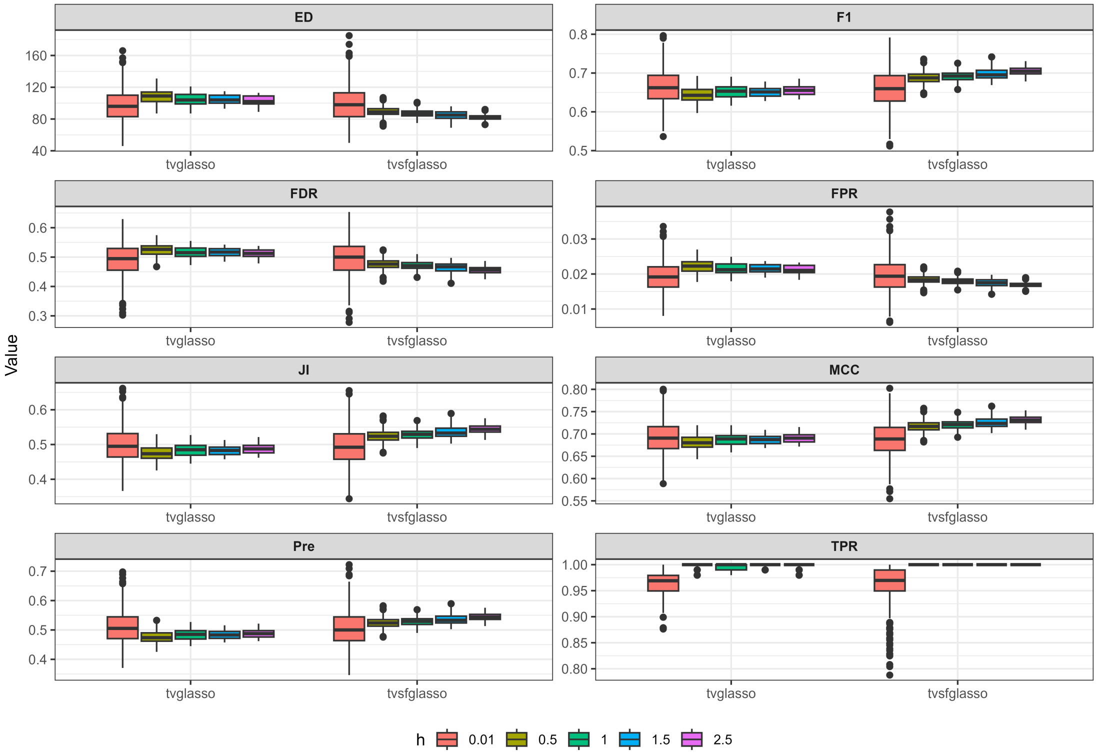


Fig H: Analysis of replicated data sets generated by simulator described in [3] using tvglasso and tvsfglasso at different values of the bandwidth parameter $h$. Different panels represent different binary classification metrics when $n = 50$.

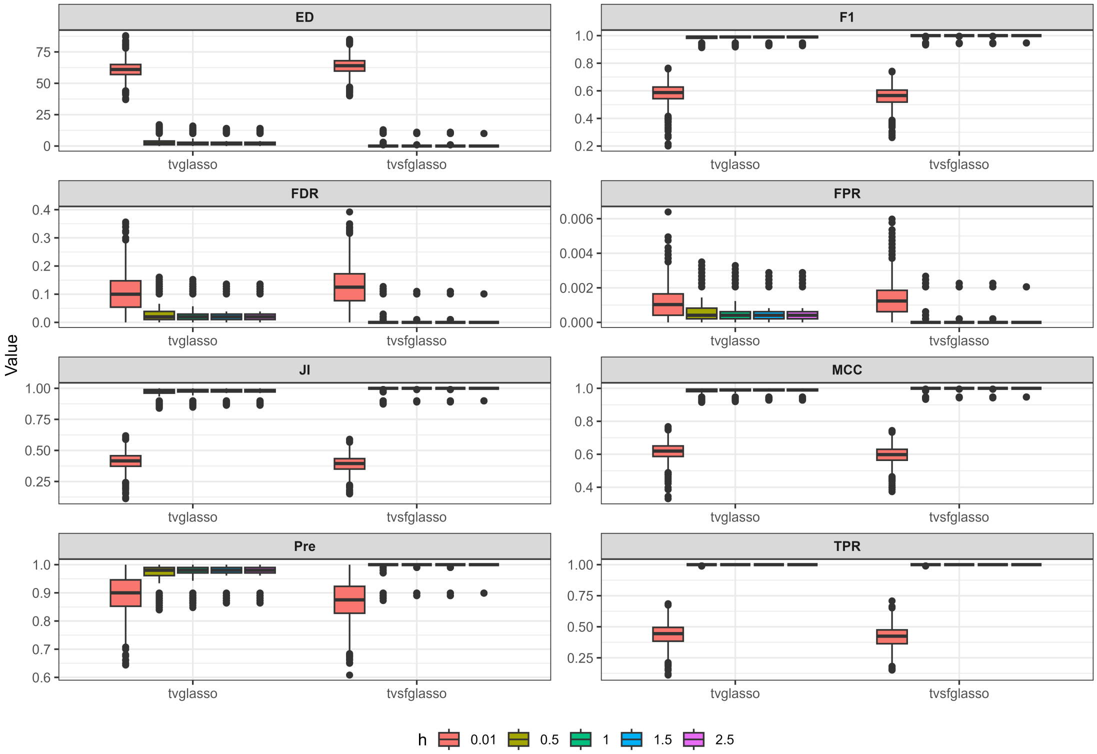


Fig I: Analysis of replicated data sets generated by simulator described in [7] using tvglasso and tvsfglasso at different values of the bandwidth parameter $h$. Different panels represent different binary classification metrics when $n = 50$.

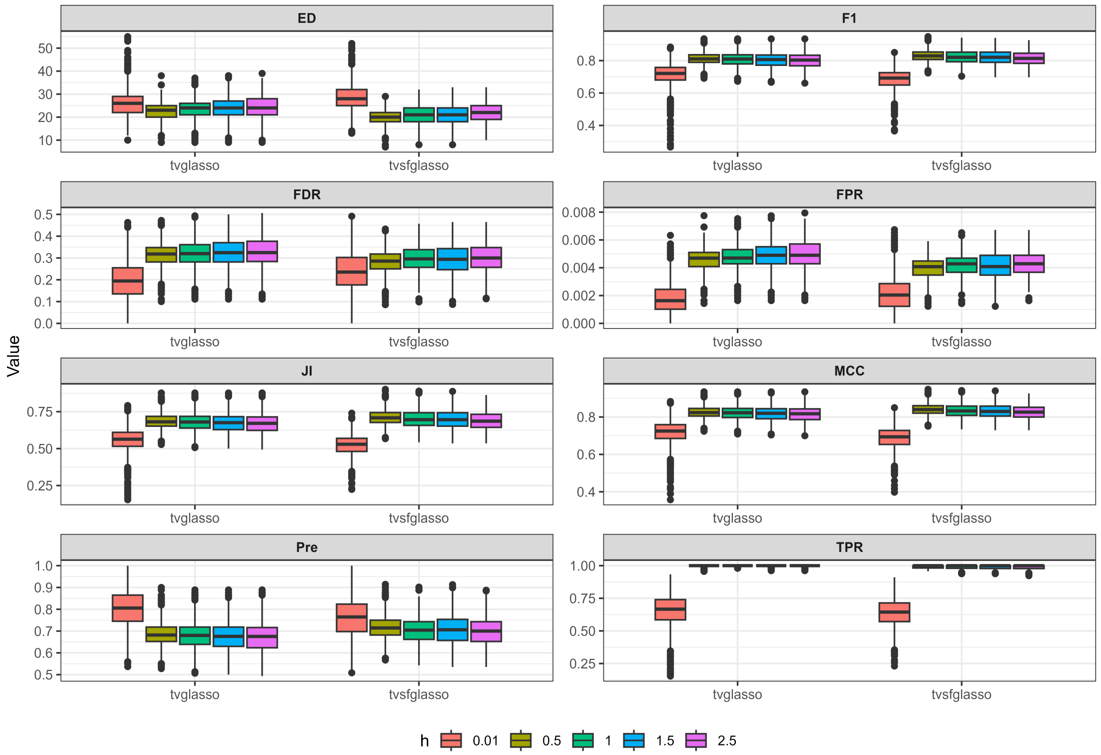


Fig J: Analysis of replicated data sets generated by simulator described in [4] using tvglasso and tvsfglasso at different values of the bandwidth parameter $h$. Different panels represent different binary classification metrics when $n = 50$.

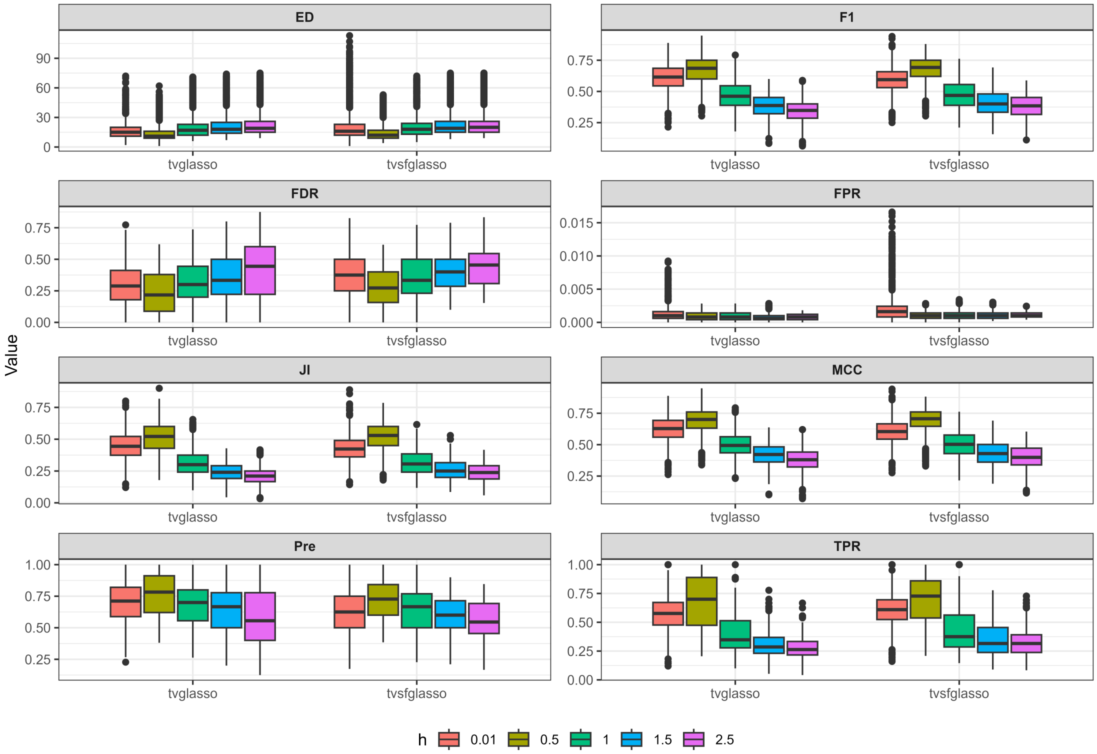


Fig K: Analysis of replicated data sets generated by simulator described in [5] using tvglasso and tvsfglasso at different values of the bandwidth parameter $h$. Different panels represent different binary classification metrics when $n = 50$.

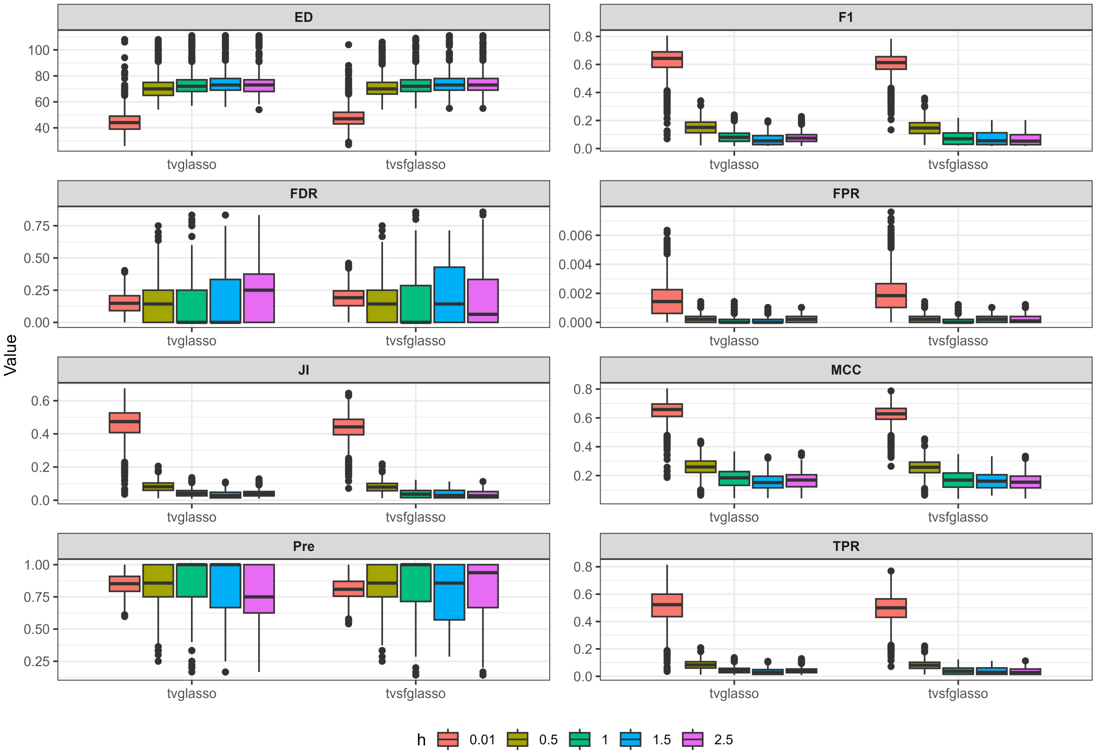


Fig L: Analysis of replicated data sets generated by simulator described in [7] (Erdős-Rényi graph) using tvglasso and tvsfglasso at different values of the bandwidth parameter $h$. Different panels represent different binary classification metrics when $n = 50$.

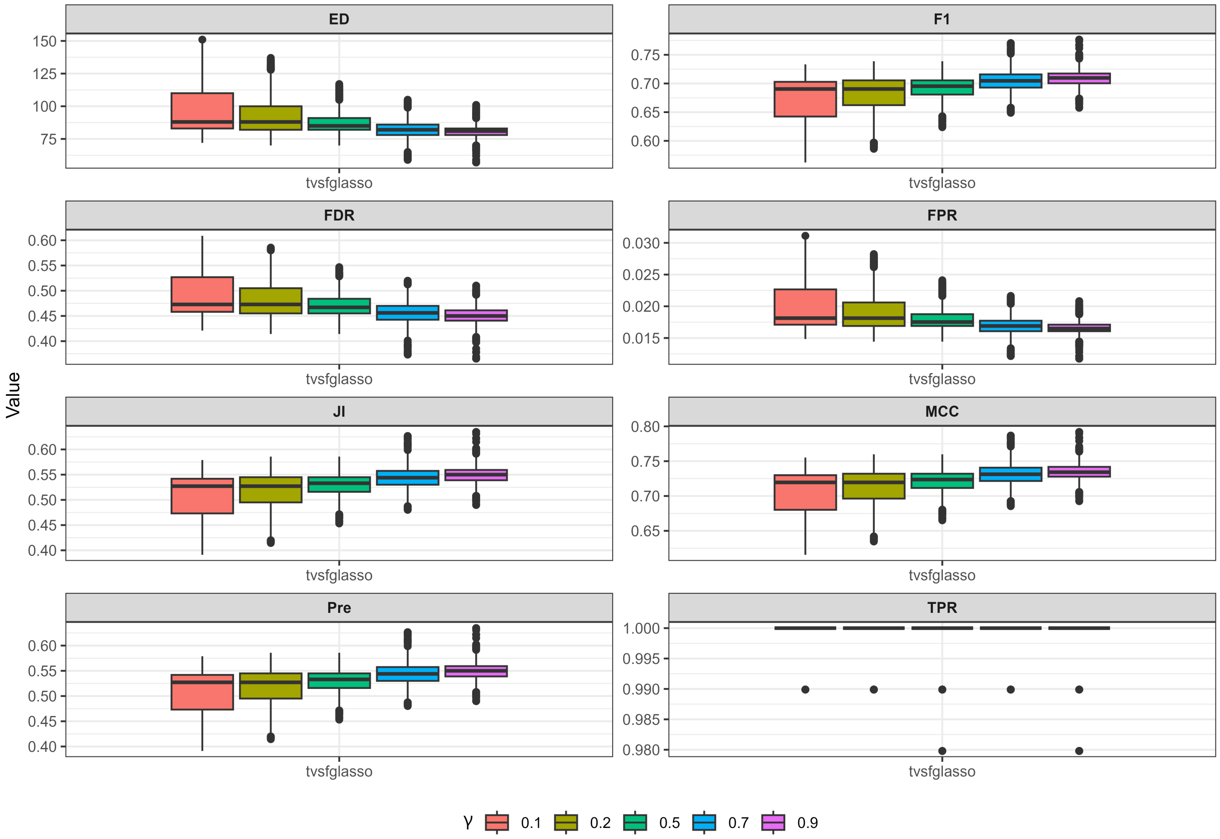


Fig M: Networks estimated at $\gamma \in \{0.1, 0.2, 0.5, 0.7, 0.9\}$, $n = 10$.

# Simulation results - eBIC $\gamma$

A sensitivity analysis of the parameter $\gamma$ of eBIC. Supplementary Figures M, N, and O illustrate the averaged results (over replicates) for time-varying scale-free glasso (tvsfglasso) at $h \in \{0.01, 0.05, 1, 1.5, 2.5\}$, $p = 100$, and $n \in \{10, 50, 100\}$.

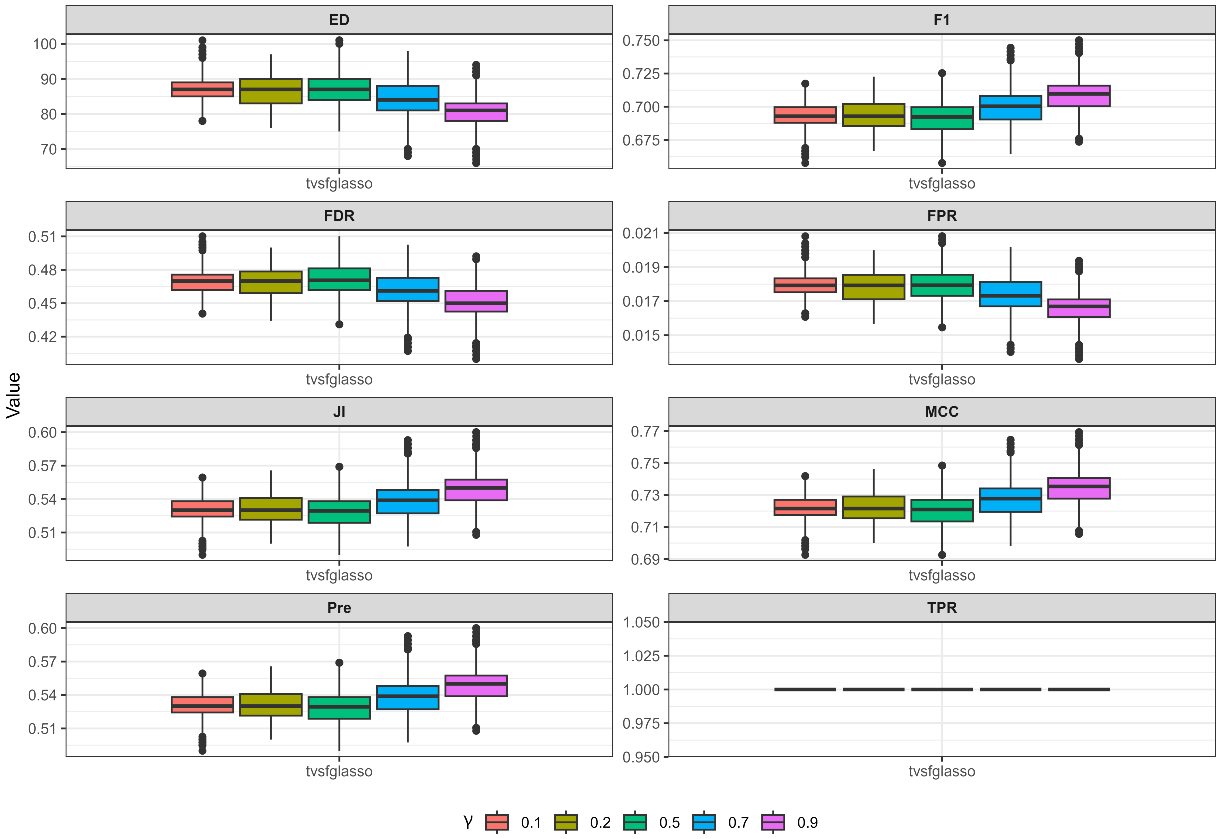


Fig N: Networks estimated at $\gamma \in \{0.1, 0.2, 0.5, 0.7, 0.9\}$, $n = 50$.

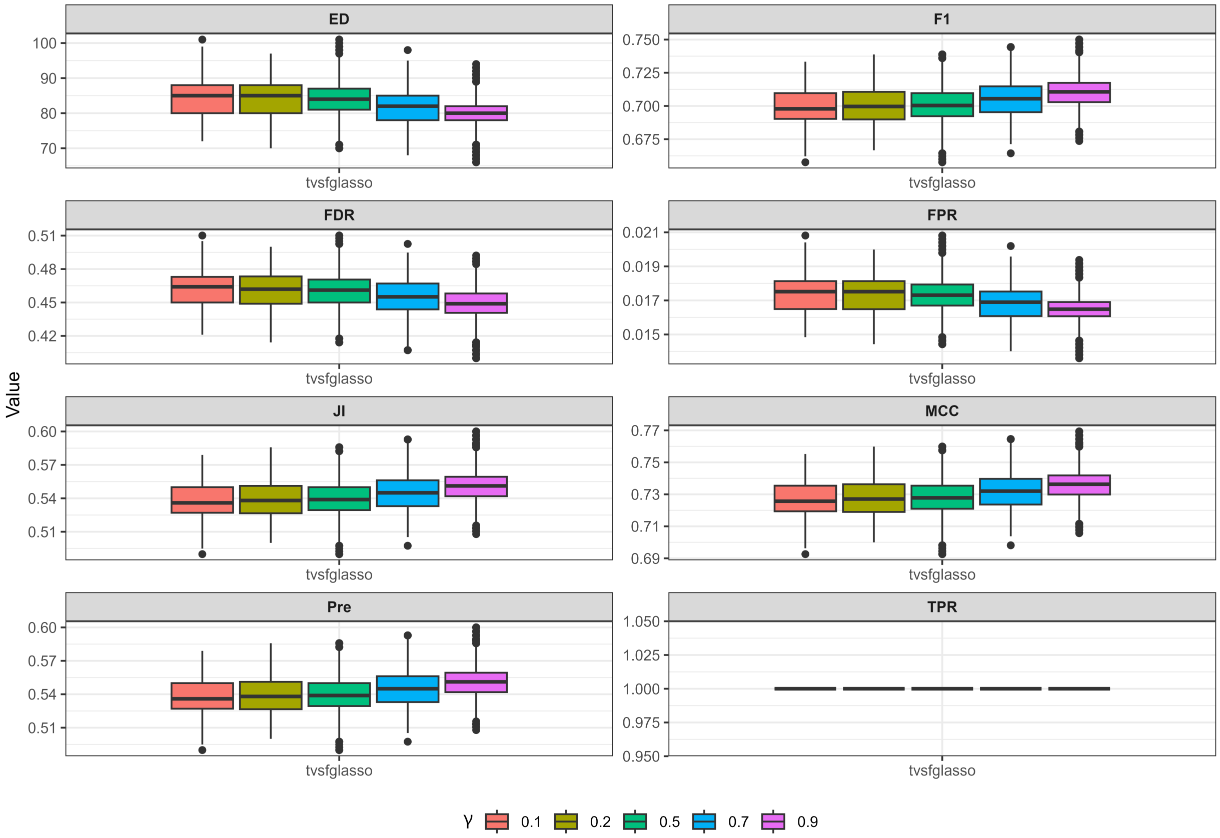


Fig O: Networks estimated at $\gamma \in \{0.1, 0.2, 0.5, 0.7, 0.9\}$, $n = 100$.

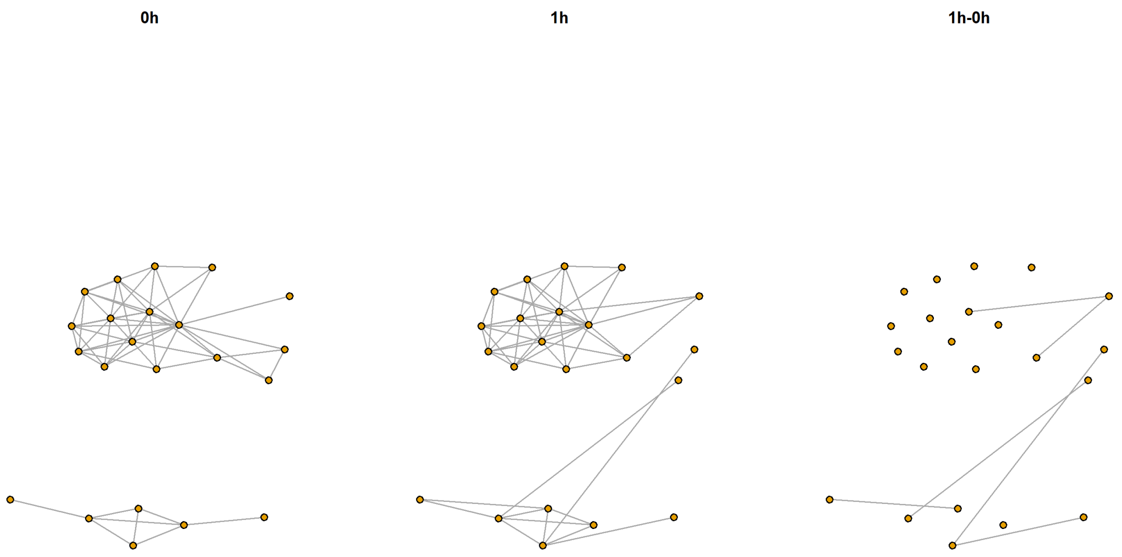


Fig P: Networks estimated at time steps 0h and 1h and their difference.

# Real data examples

Time-varying gene co-expression networks of *Drosophila melanogaster* Transcription factors. Supplementary Figures P – AB show the TF networks estimated at consecutive time steps alongside their corresponding difference graphs. Two of the greatest topological change occurs between 12 – 16 h and 16 – 20 h.

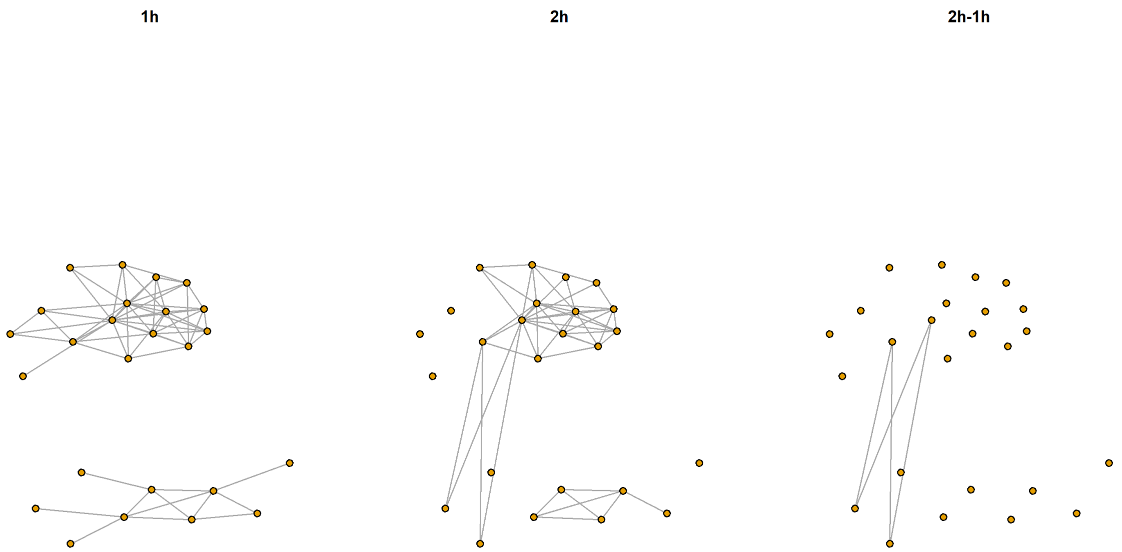


Fig Q: Networks estimated at time steps 1h and 2h and their difference.

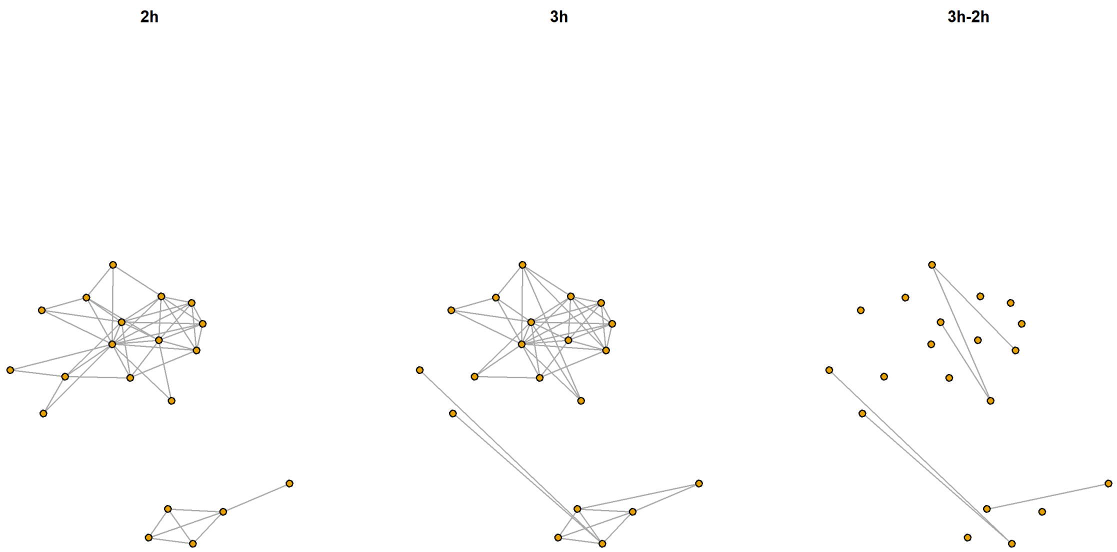


Fig R: Networks estimated at time steps 2h and 3h and their difference.

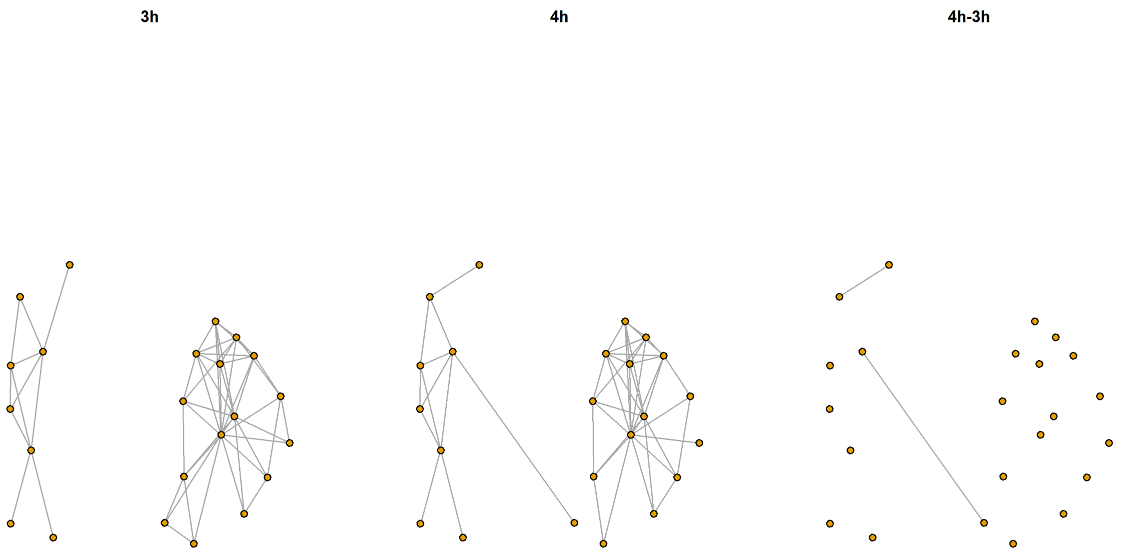


Fig S: Networks estimated at time steps 3h and 4h and their difference.

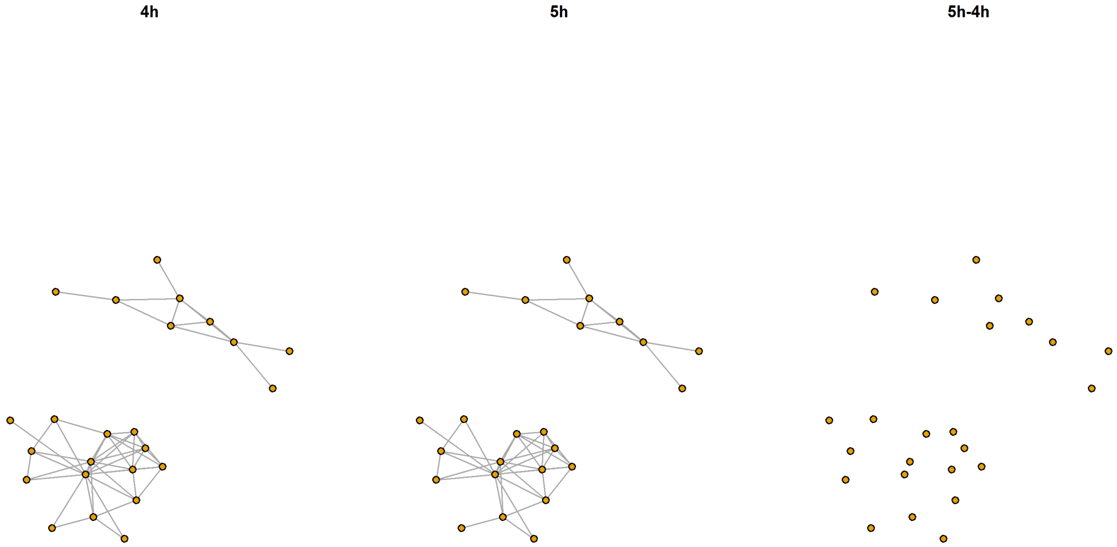


Fig T: Networks estimated at time steps 4h and 5h and their difference.

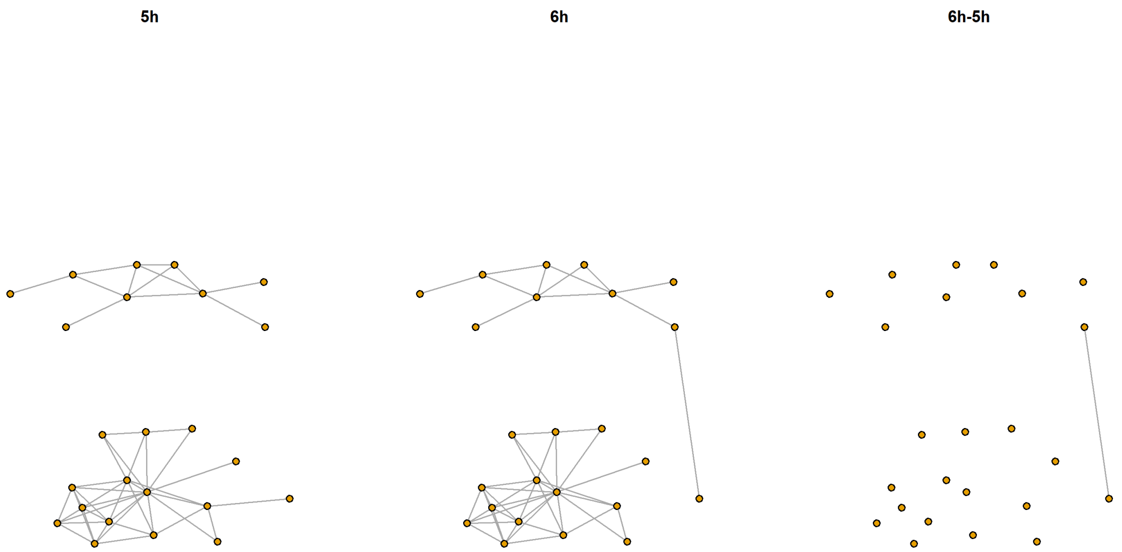


Fig U: Networks estimated at time steps 5h and 6h and their difference.

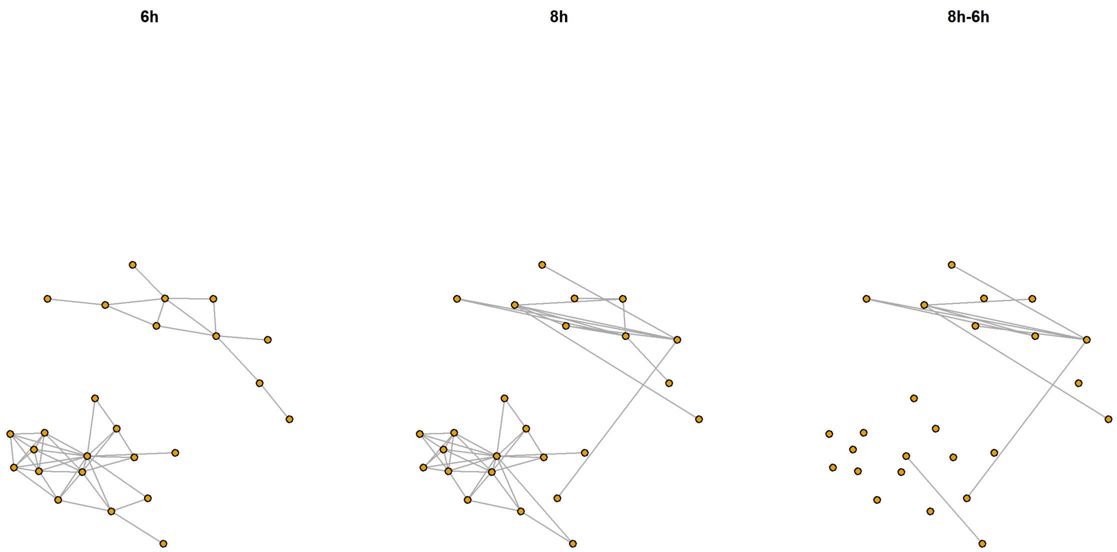


Fig V: Networks estimated at time steps 6h and 8h and their difference.

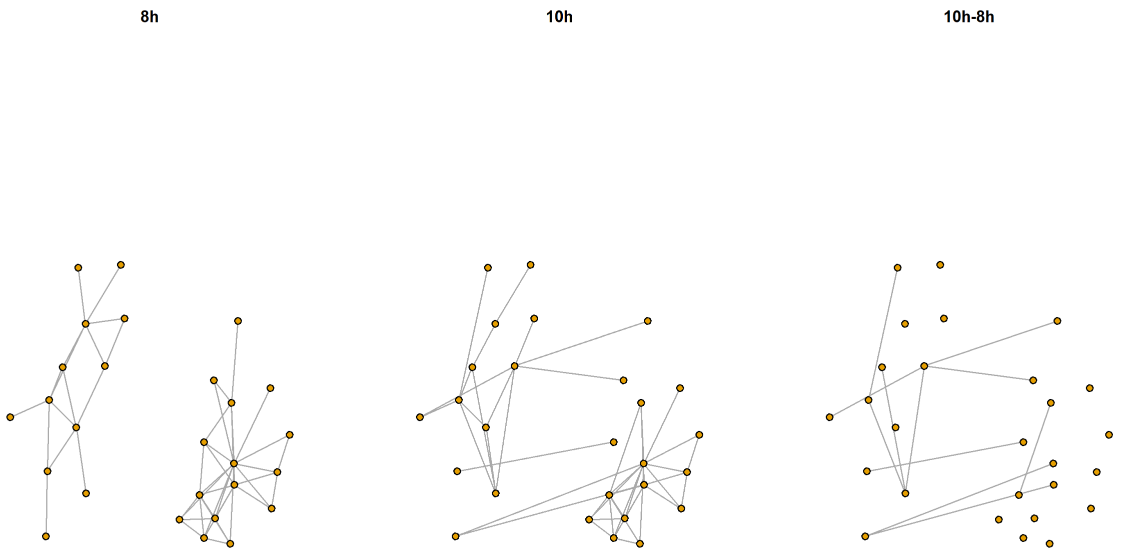


Fig W: Networks estimated at time steps 8h and 10h and their difference.

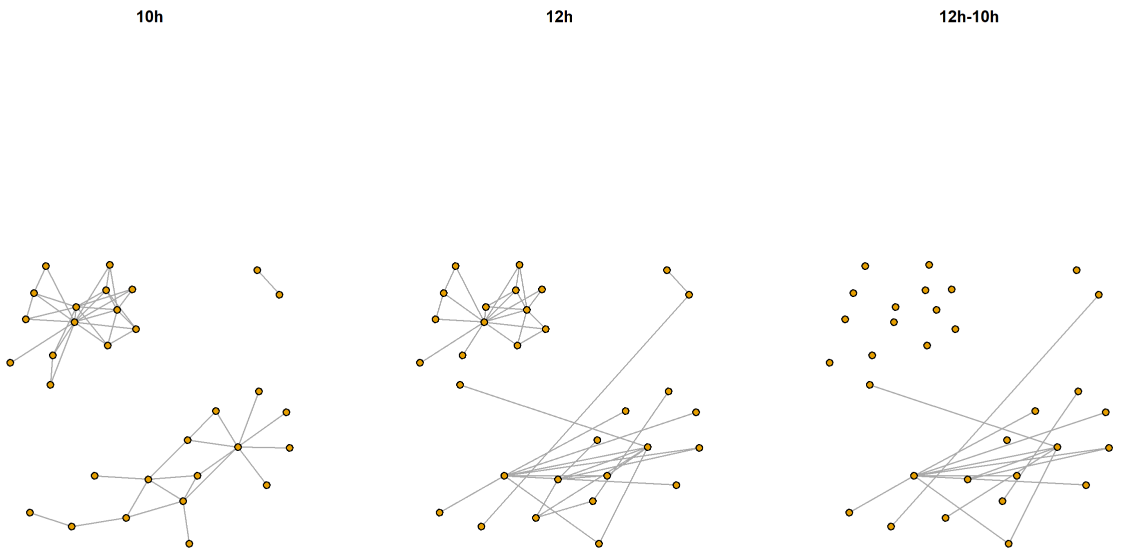


Fig X: Networks estimated at time steps 10h and 12h and their difference.

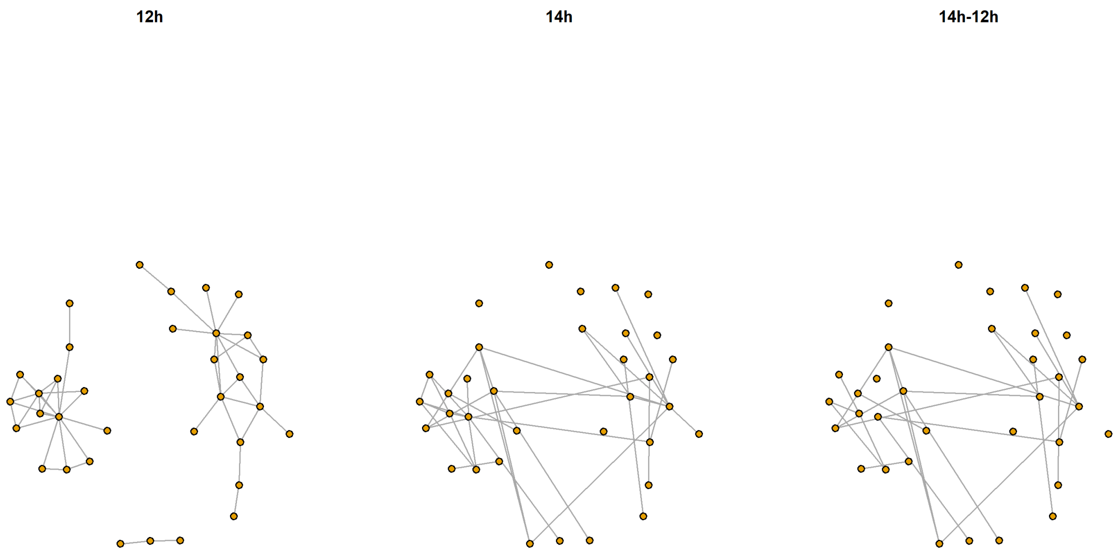


Fig Y: Networks estimated at time steps 12h and 14h and their difference.

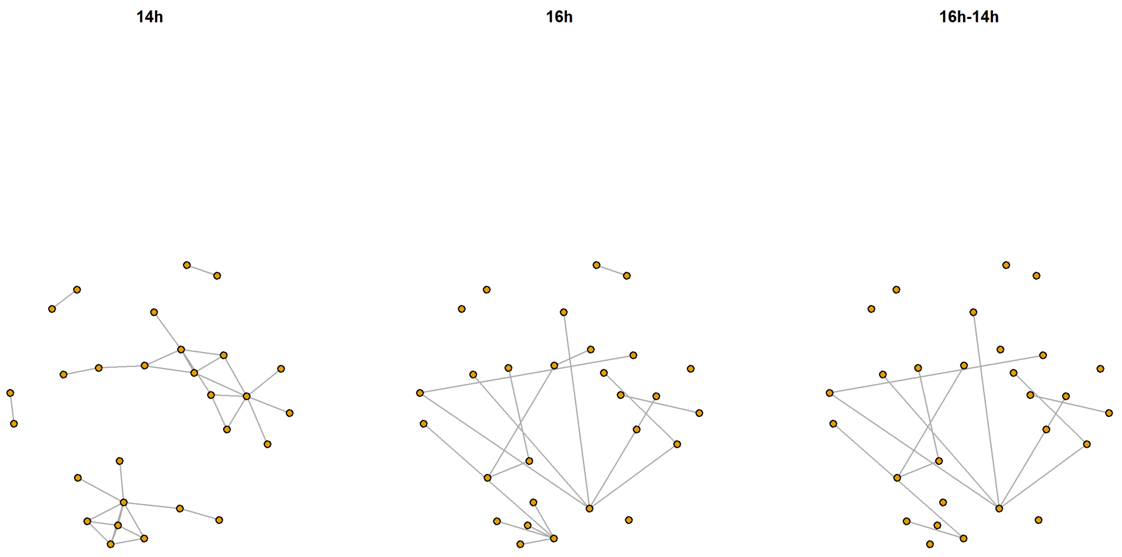


Fig Z: Networks estimated at time steps 14h and 16h and their difference.

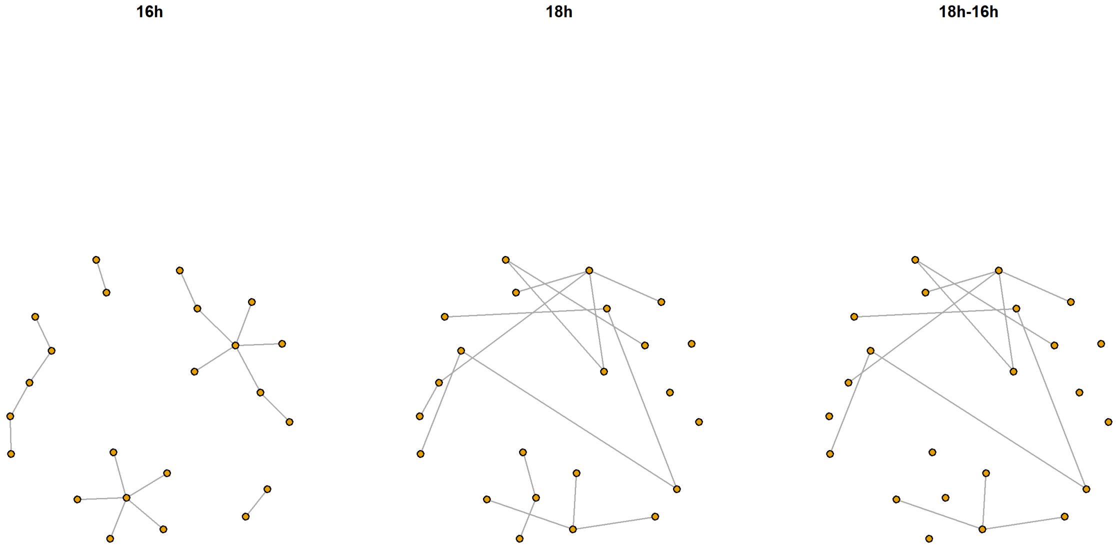


Fig AA: Networks estimated at time steps 16h and 18h and their difference.

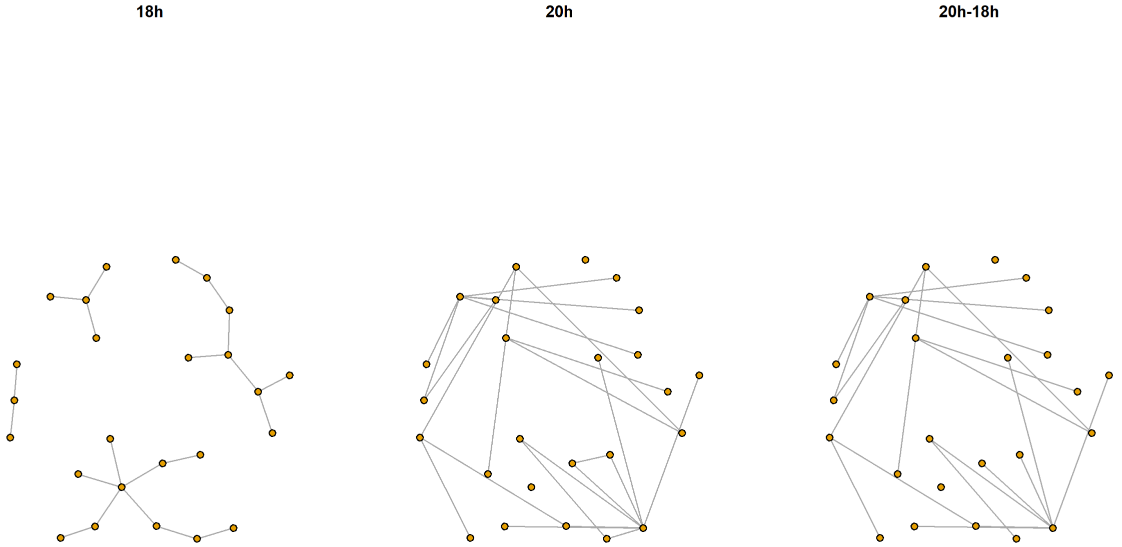


Fig AB: Networks estimated at time steps 18h and 20h and their difference.

# References


[1] Albert-Laszlo Barabási and Reka Albert. Emergence of scaling in random networks. *Science*, 286:509 – 512, 1999. https://doi.org/10.1126/science.286.5439.509.

[2] Ulrik Brandes. On variants of shortest-path betweenness centrality and their generic computation. *Social Networks*, 30:136–145, 2008. https://doi.org/10.1016/j.socnet.2007.11.001.

[3] Qiang Liu and Alexander Ihler. Learning scale free networks by reweighted $l_1$ regularization. In Geoffrey Gordon, David Dunson, and Miroslav Dudík, editors, *Proceedings of the Fourteenth International Conference on Artificial Intelligence and Statistics*, volume 15 of *Proceedings of Machine Learning Research*, pages 40–48, USA, 2011. PMLR. https://proceedings.mlr.press/v15/liu11a.html.

[4] Kean Ming Tan, Palma London, Karthik Mohan, Su-In Lee, Maryam Fazel, and Daniela Witten. Learning graphical models with hubs. *Journal of Machine Learning Research*, 15:3297–3331, 2014. http://jmlr.org/papers/v15/tan14b.html.

[5] Jilei Yang and Jie Peng. Estimating time-varying graphical models. *Journal of Computational and Graphical Statistics*, 29:191–202, 2020. https://doi.org/10.1080/10618600.2019.1647848.

[6] T. Zhao, Han Liu, K. Roeder, J. Lafferty, and L. Wasserman. The huge package for high-dimensional undirected graph estimation in R. *Journal of Machine Learning Research*, 13:1059–1062, 2012. http://jmlr.org/papers/v13/zhao12a.html.

[7] Shuheng Zhou, John Lafferty, and Larry Wasserman. Time varying undirected graphs. *Machine Learning*, 80:295–319, 2010. https://doi.org/10.1007/s10994-010-5180-0.
